# Gene expression profiling of dendritic cell tolerance dysfunction in women with systemic lupus erythematosus

**DOI:** 10.3389/fimmu.2026.1771959

**Published:** 2026-06-02

**Authors:** Ana Laura Hernández-Ledesma, Evelia Lorena Coss-Navarrete, Sofia Salazar-Magaña, Diego Ramírez-Espinosa, Lizbet Tinajero-Nieto, Estefania Torres-Valdez, Angélica H. Peña-Ayala, Guillermo Félix-Rodriguez, Gabriel Frontana-Vázquez, Jair Santiago García Sotelo, Morgane Thomas-Chollier, Gosia Trynka, Florencia Rosetti, Selene L. Fernandez-Valverde, María Gutiérrez-Arcelus, Deshiré Alpízar-Rodríguez, Alejandra Medina-Rivera

**Affiliations:** 1 Laboratorio Internacional de Investigación sobre el Genoma Humano, Universidad Nacional Autónoma de México, Santiago de Querétaro, Mexico; 2 Escuela Nacional de Estudios Superiores, Unidad Juriquilla, Universidad Nacional Autónoma de México, Santiago de Querétaro, Querétaro, Mexico; 3 Hospital General Regional No. 1, IMSS, Santiago de Querétaro, Querétaro, Mexico; 4 Hospital General Regional No. 2, IMSS, El Marqués, Querétaro, Mexico; 5 Instituto Nacional de Rehabilitación “Luis Guillermo Ibarra Ibarra”, Ciudad de México, Mexico; 6 Hospital Star Médica Querétaro, Santiago de Querétaro, Querétaro, Mexico; 7 GenomiqueENS, Institut de Biologie de l’ENS (IBENS), Département de biologie, École normale supérieure, CNRS, INSERM, Université PSL, Paris, France; 8 Group Bacterial Infection, Response and Dynamics, Institut de biologie de l’ENS (IBENS), École normale supérieure, CNRS, INSERM, Université PSL, Paris, France; 9 Open Targets, Cambridge, United Kingdom; 10 Human Genetics, Wellcome Sanger Institute, Cambridge, United Kingdom; 11 Departamento de Inmunología y Reumatología, Instituto Nacional de Ciencias Médicas y Nutrición Salvador Zubirán, Ciudad de México, Mexico; 12 School of Biotechnology and Biomolecular Sciences, The University of New South Wales, Sydney, NSW, Australia; 13 UNSW RNA Institute, The University of New South Wales, Sydney, NSW, Australia; 14 Division of Immunology, Boston Children’s Hospital, Boston, MA, United States; 15 Department of Pediatrics, Harvard Medical School, Boston, MA, United States; 16 Broad Institute of MIT and Harvard, Cambridge, MA, United States

**Keywords:** dendritic cells, immune regulation, systemic lupus erythematosus, tolerance induction, transcriptomics

## Abstract

**Background:**

Dendritic cells (DCs) are central regulators of immune tolerance, and disturbances in their phenotype and function contribute to the breakdown of self-tolerance in systemic lupus erythematosus (SLE). Tolerogenic DCs (tolDCs), which suppress autoreactive responses and promote peripheral tolerance, are a promising therapeutic focus in autoimmune diseases.

**Methods:**

Here, we analyzed the transcriptional profiles of *in vitro* generated DCs derived from monocytes of individuals with SLE and healthy controls to identify disease-specific disruptions in tolerance associated pathways.

**Results:**

Interferon stimulated genes (ISGs) emerged as dominant markers across all cellular contexts, with monocytes exhibiting the most substantial enrichment; key ISGs (I*FI27, IFI44L, USP18, IFI6*) acted as central hubs in regulatory networks, underscoring their diagnostic and pathogenic significance. In tolDCs from SLE donors, lipid metabolism pathways were selectively altered, suggesting impaired synthesis of pro-resolving lipid mediators. Additionally, diminished *IL10RA* expression and dysregulated *IRF4* activity in SLE moDCs indicated intrinsic defects in IL-10 mediated tolerogenic differentiation.

**Conclusion:**

Together, these findings suggest that interferon driven transcriptional rewiring, impaired IL-10 signaling, and aberrant lipid metabolic programming converge to compromise DCs tolerogenic capacity in SLE. This highlights key mechanistic pathways that could be targeted to restore immune tolerance and reduce chronic inflammation.

## Introduction

Systemic Lupus Erythematosus (SLE) is an autoimmune disease characterized by immune dysregulation and increased production of autoantibodies targeting several tissues and organs, leading to multisystem involvement and a wide range of clinical manifestations (1). Affecting over 3.4 million people worldwide, SLE disproportionately impacts women of reproductive age (20–45 years) and people with Asian, African, and Hispanic/Latino ancestries (2–5).

Although the precise etiology of SLE remains unclear, its development results from the interplay of genetic, epigenetic, environmental, and hormonal factors (1–6). This interplay leads to the breakdown of immune tolerance, a key mechanism that triggers atypical and hyperreactive immune responses against self-antigens (7). In healthy individuals, immune tolerance is a dynamic process that prevents inappropriate immune responses against both self-antigens and non-harmful non-self-antigens (e.g. microbiota and dietary antigens). Tolerance is maintained through well-described and coordinated central and peripheral mechanisms operating in both primary and secondary lymphoid organs. These include deletion or inactivation of self-reactive T and B cells through apoptosis, as well as peripheral regulatory mechanisms such as anergy, immune deviation, and active suppression mediated by regulatory T cells and tolerogenic antigen presenting cells (8, 9).

Dendritic cells (DCs) bridge innate and adaptive immune responses, and they can promote either immunogenic (inflammatory) or tolerogenic responses, depending on environmental stimuli (10, 11). Although DCs comprise multiple phenotypically and functionally different subsets, their role in shaping immune activation versus tolerance is particularly relevant for the context of autoimmune diseases (7). Immunogenic DCs (e.g. conventional DCs, plasmacytoid DCs or monocyte derived DCs) are specialized antigen presenting cells that capture and process antigens, undergo maturation (characterized by upregulation of co-stimulatory molecules), and produce pro-inflammatory cytokines, enhancing the activation of naïve T cells and effector adaptive responses; in contrast, tolerogenic DCs (tolDCs) exhibit lower levels of co-stimulatory molecules and higher anti-inflammatory signals, promoting immune tolerance and regulatory T cell activity (10–13).

People with SLE exhibit quantitative and functional abnormalities across multiple DCs populations, including reduced circulating conventional DCs, expansion and aberrant activation of plasmacytoid DCs, and increased differentiation of inflammatory moDCs (7, 10, 12, 14). Recent SLE studies have applied single-cell transcriptomic approaches to monocytes and dendritic cells, often using trajectory or pseudotime analyses to reconstruct differentiation pathways (15–17). While these studies have provided important insights into global interferon responses and immune heterogeneity, detailed analyses of monocyte-derived dendritic cells and tolerogenic dendritic cells in SLE context remain limited.

DCs are present in peripheral blood and constitute less than 1% of the circulating peripheral blood mononuclear cells, which makes their direct recovery for research purposes challenging (18). As a result, *in vitro* models such as *in vitro* derived monocyte derived dendritic cells (moDCs) from monocytes recovered from peripheral blood have been widely used. Although *in vitro* moDCs are not fully equivalent to *ex vivo* isolated DCs, they display hallmark functional features that make them a widely accessible *in vitro* system for studying DCs properties and functions (7). In particular, *in vitro* derived moDCs can exhibit strong proinflammatory activity and promote inflammatory T helper responses (Th1, Th2, and Th17), reflecting key aspects of *in vivo* DCs mediated immune activation, which makes them useful for studying mechanisms relevant to inflammation and autoimmune conditions (10, 14).

TolDCs have the capacity to inhibit self-reactive responses by modulating T cell activity, inducing T cell anergy, or supporting regulatory T cell (Treg) generation. They are characterized by low expression of co-stimulatory molecules (e.g., CD80, CD86, and CD83), proinflammatory cytokines and major histocompatibility complex (MHC) molecules, and elevated anti-inflammatory markers (e.g. IL-10, TGF-β, PD-L1, and PD-L2) (7, 10–12). Over the last decades, several efforts have focused on characterizing tolDCs and their capacity to restore tolerance as a therapeutic approach for autoimmune diseases such as type 1 diabetes (T1D), rheumatoid arthritis (RA), multiple sclerosis (MS), and Crohn’s disease (19). When treated with dexamethasone and rosiglitazone, moDCs from SLE patients adopted a tolerogenic phenotype with promising functional capacity to suppress T cell activation, with no significant differences found between SLE patients and healthy controls (20).

Comparing DCs subsets between SLE patients and healthy controls at a single differentiation endpoint fails to capture the dynamic and stage specific alterations that occur during monocytes to DCs differentiation processes. Analyses restricted to final phenotypes may overlook transitory but biologically relevant changes in gene expression, regulatory networks and lineage commitment processes that arise at intermediate stages of differentiation. A trajectory-based analysis of how transcriptional trajectories are implied in the generation of *in vitro* moDCs and DCs differentiated under tolerogenic conditions will enable the identification of altered differentiation kinetics, disrupted regulatory pathways, and disease-specific mechanisms involved in immune dysregulation in SLE.

The relevance of this approach is notable in Mexican populations, where SLE risk reflects a distinct genetic architecture shaped by regional admixture. Previous studies have identified genetic contributors to SLE in Mexican individuals, including variants in interferon signaling pathway genes (e.g., *TLR7*, *IRF5*), complement and adhesion molecules (e.g., *ITGAM*), TNF-related pathways, and ancestry-influenced HLA haplotypes. Despite these advances, functional genomic data from Global South populations remain limited, restricting our ability to understand population-specific altered immune cell differentiation, tolerance induction, and regulatory network dynamics (21).

## Methods

### Ethics

This study was conducted in accordance with the Declaration of Helsinki and approved by the Ethics on Research Committee of the Instituto de Neurobiología at the Universidad Nacional Autónoma de México (UNAM, Protocol 093.H), and Instituto Nacional de Ciencias Médicas y Nutrición Salvador Zubirán (IRE-3636). Written informed consent was obtained from all participants before enrollment. All clinical and demographic data was anonymized and securely stored at the Laboratorio Nacional de Visualización Científica Avanzada (LAVIS), UNAM, to ensure its confidentiality.

### Participant recruitment and sample collection

Mexican women aged between 18–50 years with SLE were identified through the Mexican Lupus Registry (LupusRGMX), part of the MEXOMICS consortium, and by certified rheumatologists (22, 23). SLE diagnosis was confirmed using the 2019 European League Against Rheumatism/American College of Rheumatology Classification Criteria (EULAR/ACR) (24). In this analysis, only participants with glucocorticoid therapy at<10 mg/day were included; however, information on the duration of the use of the current dose was not collected. We confirmed that there had been no changes in treatment over the past two months and that no biological treatment had been used in the previous six months. Exclusion criteria included pregnancy, additional chronic diseases (including other autoimmune diseases), concurrent hormonal or antibiotic treatments, use of biological treatments, and/or any modification of SLE-specific therapy in the previous two months.

In general, SLE participants were evaluated by rheumatologists, a subset of cases relied on participant self-report supported by review of their personal medical records (e.g., laboratory results and clinical notes), as electronic medical records are not consistently available in Mexico.

EDTA-anticoagulated whole blood was obtained by peripheral venous puncture from 23 Mexican women with SLE and 10 healthy controls from June 2022 to February 2023. The clinical and sociodemographic characteristics of all volunteers are summarized in **Supplementary Table 1**.

### Generation of *in vitro* moDCs and tolDCs

Peripheral blood mononuclear cells (PBMCs) were recovered using Lymphoprep™ (Stemcell Technologies) density gradient. Briefly, blood was diluted 1:1 (v/v) in PBS 1X, gently poured over Lymphoprep™ (Stemcell Technologies) and centrifuged at 3000 rpm, 20 minutes, at room temperature, without break. PBMCs were collected from the interface formed at the density gradient. Monocytes were then isolated from PBMCs using the negative selection EasySep™ Human Monocyte Isolation Kit (Stemcell Technologies) magnetic approach according to the manufacturer’s instructions. Cells were then recovered for RNA extraction and flow cytometry.

Purified monocytes were resuspended in RPMI-1640 medium supplemented with 10% FBS and seeded at a density of 1.5x106 cells per 2mL in 24-well plates. For each donor, two wells were prepared: one for *in vitro* derived monocyte-derived dendritic cell differentiation (hereinafter referred as *in vitro* moDCs) and another for dendritic cells differentiated under tolerogenic conditions (hereinafter referred as tolDCs). Initially, both wells were supplemented with 1 ng/mL of recombinant human granulocyte-macrophage colony stimulating factor (GM-CSF) (Preprotech) and 25 ng/mL of recombinant human interleukin-4 (IL-4) (Preprotech). For *in vitro* moDCs, cultures followed the supplementation with GM-CSF and IL-4 each 48 hours. For differentiation under tolerogenic conditions (tolDC), cells were cultured as described above, with daily supplementation of recombinant human interleukin 10 (IL-10; 40 ng/mL; Preprotech) starting on day 2. On day 8, cells were harvested for flow cytometry analysis and RNA extraction.

### Flow cytometry and gating strategy

The expression of surface markers associated with monocyte and DCs antigen presentation mechanisms was measured in 6 SLE and 4 control samples using flow cytometry. Cells were initially incubated with Human Fc Receptor blocking solution (TruStain FCX™, BioLegend) for 10 minutes, and then incubated with a cocktail of fluorochrome-conjugated antibodies: anti-human CD14 (myeloid cell surface marker) redFluor 710 (Clone 61D3, Tonbo Biosciences), anti-human CD11c (dendritic cell surface marker) FITc (Clone 3.9, Invitrogen), anti-human HLA-DR (dendritic cell surface marker) APC (Clone L243, Tonbo Biosciences), anti-human CD40 (dendritics cell co-stimulatory molecule] PE (Clone G28.5, Tonbo Biosciences) and anti-human CD80 (dendritics cell co-stimulatory molecule) PerCP-Cy 5.5 (Clone 2D10, Tonbo Biosciences). Staining was performed for 30 minutes at 4 °C, in the dark.

The gating strategy is detailed in **Supplementary Figure 1**. Briefly, we gated on the total cell population by forward scatter area (FSC-A) versus side scatter area (SSC-A) to exclude debris. Singlets were selected by gating on FSC-H vs. FSC-A to remove doublets, as recommended in flow cytometry guidelines. Within the singlet population, cells positive for both CD14 and CD11c were identified, defining the myeloid/monocyte-DCs compartment. We then measured the expression of HLA-DR, CD40, and CD80 within this CD14^+^CD11c^+^ gate to assess DCs maturation and antigen-presenting potential. Data was recovered and analyzed using FlowJo v7.6.2 and visualized in R using the ggplot2 package.

### RNA isolation and library preparation

Recovered cells were resuspended in 200 μL of RNAlater and stored at -20 °C up to extraction. Cells were resuspended in 1 mL of PBS 1X and centrifuged for 7 minutes at 14,000 rpm and 4 °C and then resuspended in 500μL of Trizol reagent (TRIzol^®^ RNA Isolation Reagent, Invitrogen) and stored at -70 °C for the night. Total RNA was extracted using RNeasy Mini Kit (Qiagen) according to the manufacturer’s instructions. Samples were treated with DNase I (TURBO DNA-free™ Kit Invitrogen) to eliminate any residual DNA. RNA concentration, quality and integrity was assessed using the RNA high sensitivity kit (Qubit™ RNA HS Assay Kit, Invitrogen) and the Qubit 4 Fluorometer (Invitrogen). Samples were stored in RNAse-free water at -20 °C until sequencing.

RNA sequencing was performed by Novogene Inc. (Sacramento, CA, United States) using their mRNA-seq service. Libraries were prepared by poly A enrichment, and sequencing was performed on the Illumina NovaSeq X Plus^®^ system in a 150 bp paired-end format. Details on all transcriptomes generated in this study are provided in **Supplementary Table 2**.

### Differential expression analysis

RNA-seq data was analyzed using Nextflow (v23.04.3) in combination with Singularity (v3.7.0). Preprocessing of raw data was performed with the nf-core/RNAseq pipeline (v.3.14.0; https://github.com/nf-core/rnaseq, SciLifeLab) using GRCh38/hg38 (RefSeq accession GCF_000001405.26; https://www.ncbi.nlm.nih.gov/datasets/genome/GCF_000001405.26/) downloaded from UCSC as reference genome. Quality control and adapter trimming were carried out with Trim Galore! (https://github.com/FelixKrueger/TrimGalore), while alignment and transcript quantification were conducted with the STAR–Salmon workflow (25).

Quantification results from Salmon were imported into R using the tximport package (v1.18.0) (26). To explore global variations in gene expression profiles between the experimental groups (SLE and control), raw counts were transformed using a variance stabilization approach (VST) implemented in the DESeq2 package (v1.30.1) (27) and Z-score normalized. A Principal Component Analysis (PCA) was applied to the normalized expression matrix, and the first two principal components were selected for visualization (**Supplementary Figure 2**). No batch effects were observed between SLE vs control, and date of processing (**Supplementary Figure 2A, B**).

To identify differentially expressed genes (DEGs), the DESeq2 package (v.1.30.1) (27) was used on the raw count matrix, applying the Wald test for pairwise comparisons. No expression-based filtering was applied prior to differential expression testing to retain low-abundance transcripts; however, interpretation focused on statistically supported genes and pathway-level enrichment. Multiple testing correction was conducted, within each contrast independently, by controlling the false discovery rate (FDR) at 0.05 using the Benjamini–Hochberg (BH) procedure (28). We defined differentially expressed genes as those with an absolute log2FoldChange (LFC) greater than 0.5 and an adjusted p-value (padj)< 0.05. To address our different questions, the following pairwise comparisons were applied:

1. Comparisons between SLE vs. controls within each cell type: DEGs were identified by comparing SLE patients to healthy controls separately in each cell type (monocytes, *in vitro* moDCs, and tolDCs). This approach allowed the identification of cell type-specific transcriptional signatures associated with SLE. The model employed was ~ Group in each cell type.
2. Comparison of monocyte to DC differentiation trajectories between SLE and non-SLE controls: We characterized transcriptional changes associated with monocyte to dendritic cell differentiation, independent of disease group. *In vitro* moDCs and tolDCs were compared to monocytes separately within the SLE and control groups. This analysis provided baseline differentiation signatures in healthy controls and revealed how these programs were altered in SLE patients. The model employed was ~ Group + Cell_type.
3. SLE interaction effect on the *in vitro* moDCs and tolDCs differentiation: This interaction analysis highlighted SLE-specific effects on monocyte-to-DCs transcriptional programs. The model employed was ~ Group + Cell_type + Group: Cell_type.

To identify differentially expressed genes shared with the differentiation analysis results (comparison 2) or unique to this interaction dataset, we merged the tables using the full_join or left_join function from the dplyr package in R (v1.1.4).

### Gene ontology and pathway enrichment analysis

For each set of differentially expressed genes of interest, functional enrichment analysis was performed using g:Profiler2 (v0.2.0) (29), together with supporting R packages: enrichplot (v1.10.2) (30), DOSE (v3.16.0) (31), clusterProfiler (v3.18.1) (32, 33), and tidyverse (v2.0.0) (34). Genes were queried against multiple annotation databases, including Gene Ontology (GO: Biological Process, Cellular Component, Molecular Function), Reactome (REAC), Human Phenotype Ontology (HP), KEGG, WikiPathways (WP), transcription factor (TF) targets, CORUM protein complexes, and miRNA annotations. Significance was assessed using the hypergeometric test, with multiple testing corrections applied through the FDR and BH procedure. Only terms with padj< 0.05 were considered significant.

### Regulatory network analysis

To explore gene regulatory mechanisms, we used pySCENIC’s (https://github.com/aertslab/pySCENIC) “scenic_multiruns” pipeline implemented by VSN-Pipelines (v0.25.0) (35), executed with Nextflow (v23.04.3) in combination with Singularity (v3.7.0). This enabled the construction of protein–protein interaction, co-expression, and competitive endogenous RNA (ceRNA) networks, as well as to infer regulatory modules. The input was the VST-transformed gene expression count matrix obtained using DESeq2 (v.1.30.1) (27). We filtered regulons (defined as transcription factors and their associated target genes) by retaining those detected in at least forty runs across 100 iterations, with target genes required to be assigned to the same transcription factor in at least five independent runs. A lower recurrence threshold than typically used (e.g., 80 runs) was applied because our analysis focused on a restricted subset of the regulatory landscape centered in differentially expressed genes.

Regulon activity matrices of AUC (area under the recovery curve) values were extracted from the resulting SCENIC’s loom file and matched to the corresponding sample metadata. To determine differential regulons, we first evaluated overall AUC normality using the Shapiro–Wilk test. Regulons with normally distributed AUC values were tested using an unpaired Student’s t-test, whereas non-normal regulons were evaluated with a Mann–Whitney U test. Multiple testing correction was done using the Benjamini–Hochberg method in Python (v3.8.19) and the SciPy package (v1.10.1). Log_2_fold change (log_2_FC) was computed per regulon from sample-wise mean AUC values. We deemed a regulon differential if the adjusted p-value was< 0.05 and the absolute log_2_FC was > 0. The analyses were performed following the steps outlined below:

1. To identify regulons significantly altered in SLE, we compared AUC values from SLE samples to those from control samples within each cell type, using controls as the reference group.
2. To assess changes along the monocyte-to-DC differentiation trajectories, we compared *in vitro* moDCs AUC values to those of monocytes, and tolDCs AUC values to those of monocytes, using monocytes as the reference in both SLE and control samples. Then we subset the target genes of SLE-exclusive regulons that also showed differential expression in comparison 2.

To identify patterns that could be shaping the global behavior of SLE-derived *in vitro* moDCs and tolDCs separate from controls, we first subset the AUC values (z-score normalized) to the regulons that target shared DEGs between *in vitro* moDCs and tolDCs, from the interaction analysis (comparison 3). For each regulon j, we removed the effect of the cell type (*in vitro* moDCs or tolDCs) by fitting a linear model of the form:


$$R_{ij}=\beta_{0j}+\beta_{1j}\cdot I\left(c_{i}=tolDC\right)+\epsilon_{ij}$$

Where:

- $R_{ij}$ denotes the regulon j activity (Z-score) for sample i,
- $B_{0j}$ is the mean regulon activity on *in vitro* moDCs,
- $B_{1j}$ represents the average difference between tolDCs and *in vitro* moDCs,
- I(·)  is a binary indicator variable equal to 1 for tolDCs and 0 for *in vitro* moDCs.
- $c_{i}$ represents the cell type c for each sample i.
- and $\epsilon_{ij}$ represents the residuals.

To reveal variation not explained by cell type, we extracted a matrix of residuals and performed PCA on those values. Then, to evaluate each regulon’s contribution, we compared the absolute mean Z-scores between SLE and controls, prioritizing the regulon with the largest absolute mean difference.

### Statistical analyses and data visualization

Data and statistical analyses of the clinical and sociodemographic data were performed using R (v.4.0.2) (36). The normal distribution of sociodemographic and clinical data was evaluated using the Shapiro-Wilk test. For variables with normal distribution, the mean and standard deviation (SD) were reported, while for variables with a non-normal distribution, the median, interquartile range (IQR), minimum, and maximum values were calculated. Since age presented a normal distribution, the student’s t-test was used to compare values between SLE and controls. These tests were performed using the “stats” package (v.3.6.2).

Additional enrichment analyses were evaluated using the hypergeometric over−representation test implemented in phyper, base package in R, and a one−tailed Fisher’s exact test implemented in fisher.test from stats package in R.

All figures including heatmaps, scatterplots, box plots, barplots, networks and upset plots were created using a combination of ggplot2 (v3.5.1) (37), ggh4x (v0.2.8) (38), gridExtra (v2.3) (39), circlize (v0.4.16) (40), reshape2 (v1.4.4) (41), tidyverse (v2.0.0), ComplexHeatmap (v2.20.0) (34) and Cytoscape (42).

## Results

### Overview of the experimental workflow

**Figure 1:**
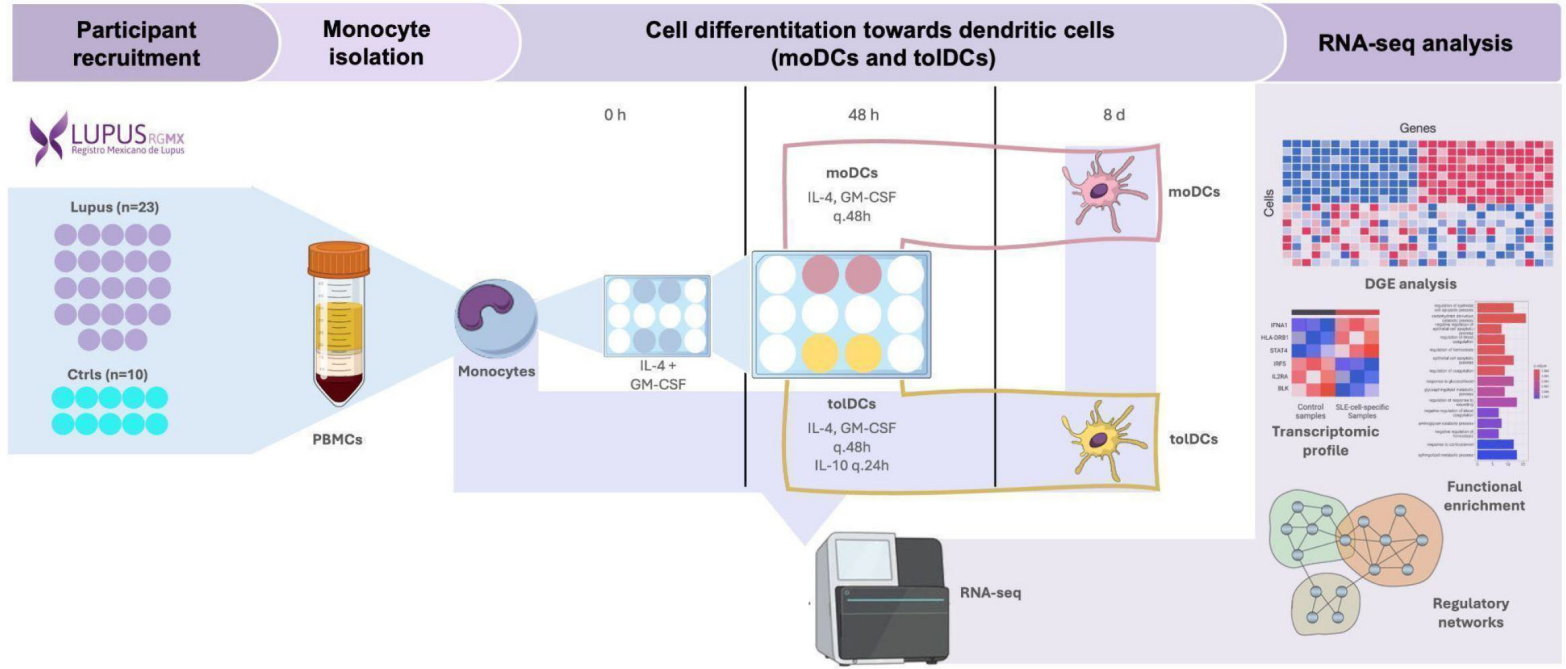
Experimental design of the study. Schematic overview of the experimental workflow. Blood samples were collected from volunteers recruited through LupusRGMX. Peripheral blood mononuclear cells were isolated, and monocytes were subsequently purified and differentiated *in vitro* into moDCs (pink cells) or tolDCs (yellow cells). Monocytes, *in vitro* generated moDCs and tolDCs were harvested for total RNA extraction, and high-throughput RNA sequencing was performed. Downstream bioinformatic analyses included differential gene expression (DGEs), functional enrichment, and regulatory network.

Peripheral blood samples were obtained from 23 Mexican women with SLE and 10 healthy controls recruited through LupusRGMX and certified rheumatologists. All SLE diagnoses were confirmed using the 2019 EULAR/ACR classification criteria (24), and participants were selected to minimize treatment-related confounding, including stable low dose glucocorticoid use and absence of recent biologic therapy (See Methods). PBMCs were isolated from each sample, and monocytes were purified for immediate analysis or differentiated *in vitro* into moDCs or tolDCs using established cytokine stimulation protocols (See Methods). Monocytes, *in vitro* moDCs, and tolDCs were then harvested for flow cytometric characterization and RNA sequencing, followed by differential expression, functional enrichment, and regulatory network analyses (**Figure 1**).

### Clinical characterization of the cohort

We initially calculated descriptive statistics and assessed data distribution for all continuous variables (histograms, Q-Q plots, and Shapiro–Wilk tests; (see **Supplementary Figure 3**; **Supplementary Table 3**), before summarizing sociodemographic and clinical characteristics. We recruited 23 volunteers with SLE and 10 healthy controls from LupusRGMX (sociodemographic and clinical characteristics of the volunteers are presented in **Table 1**). Volunteers with SLE had a mean age (± SD) of 33.7 (± 8.6) years, whereas the healthy control group had a mean age (± SD) of 29.9 (± 7.0) years. SLE participants reported a median (IQR) age at diagnosis of 27.0 (IQR: 16.0) years, with a median (IQR) of 5.7 (IQR: 4.7) years since the diagnosis. Six (26.1%) of the participants reported a family history of SLE and three (13%) had been diagnosed with lupus nephritis (**Supplementary Tables 1**, **S4**). Regarding SLE activity, we observed that ten volunteers (43.5%) had disease activity. As for treatment consumption, sixteen (69.6%) of the participants use corticoids on a regular basis, with a median daily dose of 5 mg. The use of antimalarials was also frequently reported by our cohort (78.3%).

**Table 1:** Sociodemographic and clinical characteristics of the volunteers with SLE (n=23) and Ctrl (n=10).

Characteristic	SLE (n=23)
Age (years)	Mean ± SD: 33.7 ± 8.6
Time with diagnosis (years)	Median (IQR): 4 (4.5) Min-Max: 1-17
Age at diagnosis (years)	Median (IQR): 27 (16) Min-Max: 16-43
Disease activity (n,%)	
Without activity/low activity (0–5 pts)	13 (56.5%)
With activity (≥6 pts)	10 (43.5%)
Nephritis (n,%)	3 (13.0%)
Family history of SLE (n,%)	6 (26.1%)
Treatment use (n,%)	
Glucocorticoids use *Dose (mg/day)* Antimalarials Mycophenolate Azathioprine Methotrexate Rituximab Others	16 (69.6%) Median (IQR): 5 (0) Min-Max: 2.5-8 18 (78.3%) 7 (30.4%) 6 (26.1%) 2 (8.7%) 1 (4.3%) 5 (21.7%)

SD, Standard Deviation; IQR, Interquartile Range.

### Immune phenotype variability in SLE-derived DCs

To evaluate the effects of our different supplementation conditions on cell phenotypes, we performed flow cytometry on all cell types (**Supplementary Figure 1**). We first quantified the percentage of CD14^+^ (myeloid cell surface marker) and CD11c^+^ (dendritic cell surface marker) cells, observing that monocytes had a lower frequency of CD14^+^CD11c^+^ cells (median 0.8%, IQR 11.0%) compared to *in vitro* moDCs (median 34.9%, IQR 15.4% and tolDCs (median 42.9%, IQR 32.8%). Notably, the proportion of CD14^+^CD11c^+^ cells was reduced in the SLE group relative to controls across monocytes (median SLE 0.03% vs. Ctrl 15.1%, *p* = 0.0105), moDCs (median SLE:27.3% vs. Ctrl: 59.9%, *p=*0.0190), and tolDCs (median SLE 24.25% vs. Ctrl 61.0%, *p* = 0.0190). HLA-DR (dendritic cell surface marker) was highly expressed across all samples (median 94.7%, IQR 10.85). SLE-derived *in vitro* moDCs showed a slightly broader, still non-significant, distribution (variance SLE = 395.7, variance Ctrl = 111.7; Levene’s test p = 0.4804). Similarly, *CD40* (dendritic cell surface marker) expression was high across all samples (median 98.9%, IQR 3.3). The distribution of *CD40* expression in SLE-derived *in vitro* moDCs was moderately broader than in controls, but the difference did not reach significance (variance SLE = 209.3, variance Ctrl = 15.1; Levene’s test p = 0.3494). Together, these patterns for *HLA-DR* and *CD40* in SLE-derived moDCs are consistent with greater heterogeneity in SLE-derived cells. *CD80* (dendritic cell co-stimulatory molecule) also exhibited a high expression among all samples (median 91.2%, IQR 11.2%), with SLE samples displaying a slightly wider distribution than controls. The proportion of CD80^+^ cells was reduced in the SLE group relative to controls across monocytes (median SLE: 92.1% vs. Ctrl: 85.1%, p=0.7620); whereas it was increased in *in vitro* moDCs (median SLE: 88.2% vs. Ctrl: 92.7%, p=0.6100), and tolDCs (median SLE: 90.4% vs. Ctrl: 92.5%, p= tolDCs: 1.0000).

**Figure 2:**
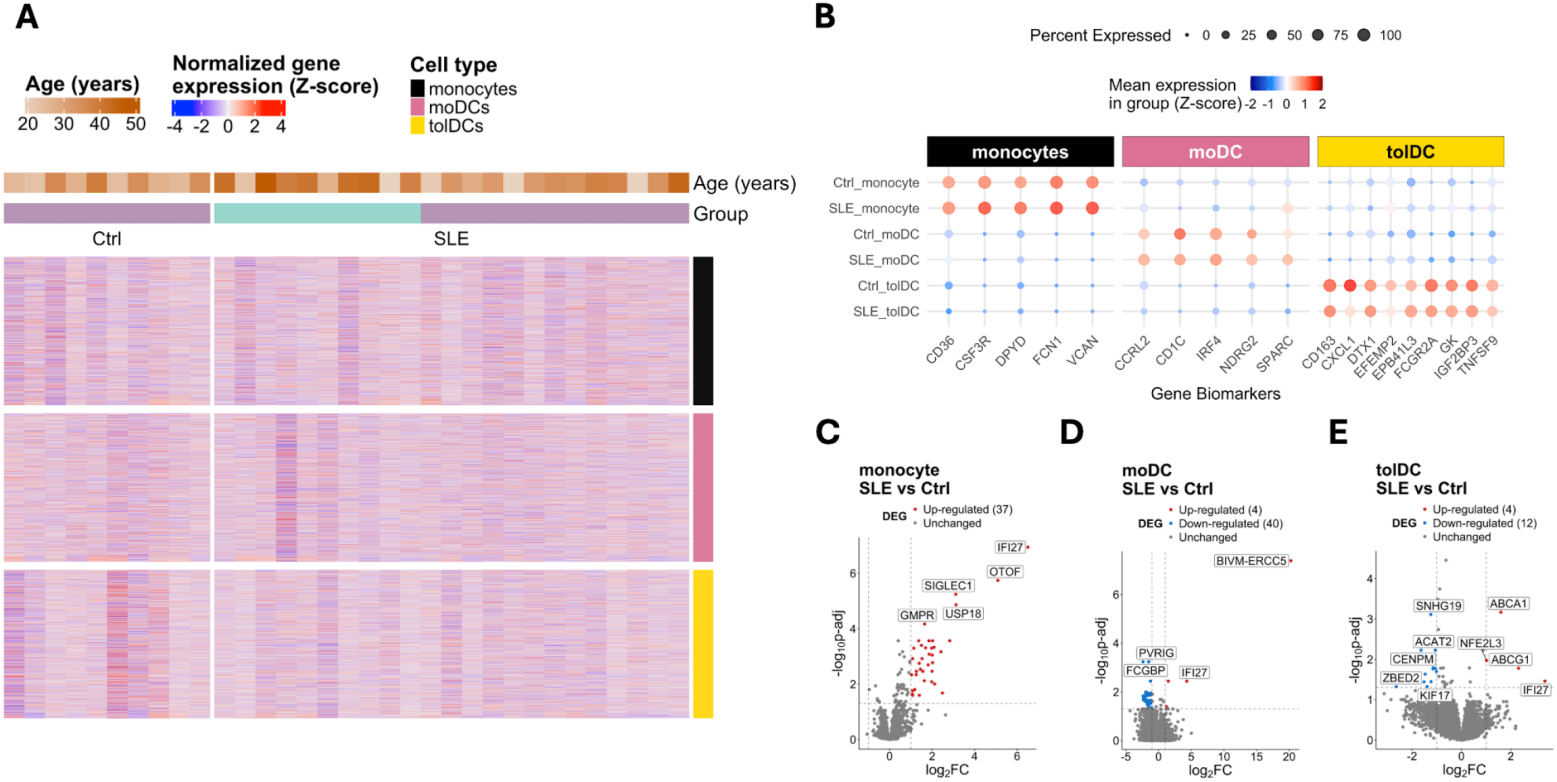
Cell type validation and integrated transcriptomic profiling across disease groups. **(A)** Heatmap of normalized gene expression, with genes represented in rows and samples in columns. Samples include monocytes, *in vitro* moDCs and tolDCs. Color scale represents Z-score-normalized counts. Metadata for each sample is annotated at the top: “Group” denotes the disease status of each sample (SLE patient and Ctrl), **(B)** Cell type-specific biomarkers. Color scale indicates average expression per group (z-score normalization); dot size reflects the percentage of samples expressing each marker. Each column is a different biomarker gene for the cell types annotated at the top. Volcano plots of differentially expressed genes in **(C)** monocytes, **(D)** *in vitro* moDCs, and **(E)** tolDCs between groups, selection was based on LFC ≥ 0.5 and padj< 0.05, using the Wald test with Benjamini-Hochberg correction for FDR.

To validate the expected cell types, we *a priori* selected established cell type–specific markers based on previous literature (**Table 2**), prior to performing differential expression analyses. We then assessed the expression of these genes across all samples. We observed the expected upregulation of cell type-specific markers in their respective cell type samples, confirming transcriptionally that the *in vitro* differentiation protocols were successful (**Figure 2**).

**Table 2:** Genetic markers of cell identity.

Cell type	Genes associated	References
Monocytes	*CD36*	(43)
	*CSF3R*	(44)
	*DPYD*	(45)
	*FCN1*	(46)
	*VCAN*	(45–47)
moDCs	*C1orf115*	(48)
	*CCRL2*	(49)
	*CD1C*	(50–52)
	*IRF4*	(51, 52)
	*NDRG2*	(46)
	*PTGDS*	(53)
	*SPARC*	(54)
tolDCs	*CD163*	(48)
	*CXCL1*	(12)
	*DTX1*	(55)
	*EFEMP2*	(12)
	*EPB41L3/FBLN4*	(48)
	*FCGR2A/CD32*	(46)
	*GK*	(48)
	*IGF2BP3*	(48)
	*TNFSF9/4-1BBL/CD137L*	(56, 57)

### Cell type-specific transcriptional signatures of SLE

**Figure 3:**
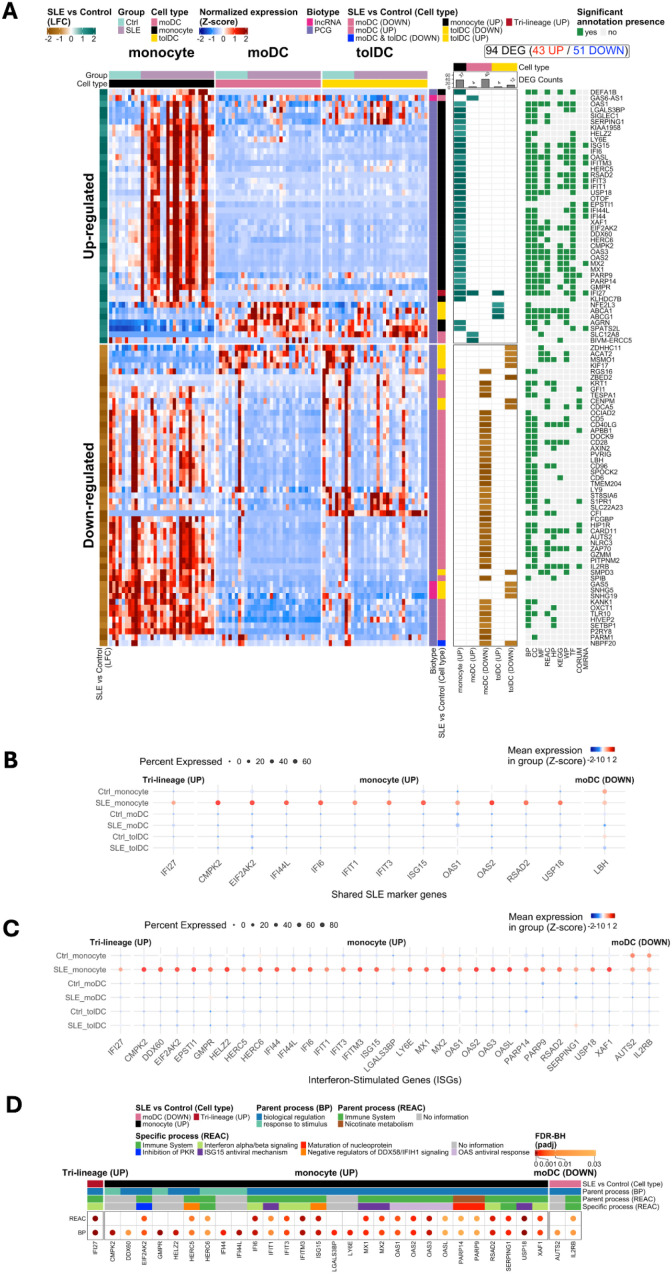
Functional enrichment analysis reveals cell type specific perturbations and a shared SLE mark. **(A)** Integrated visualization of DEGs and functional enrichment in SLE. Left panel: Heatmap of 57 DEGs (26 upregulated and 31 downregulated) between SLE and Ctrl groups across cell types, selected using LFC ≥ 0.5 and padj< 0.05. Color scale corresponds to Z-scores. Middle panel: Heatmap showing log_2_FoldChange values for SLE versus Ctrl (brown to green) across cell types. Each column includes a barplot indicating the number of DEGs per cell type: 20 mo (up), 4 *in vitro* moDCs (up), 29 *in vitro* moDCs (down), 4 tolDCs (up), and 12 tolDCs (down). Right panel: Heatmap of functional terms enriched among DEGs, identified using g:Profiler2 across multiple databases (GO : BP, CC, MF, REAC, HP, KEGG, WP, TF, CORUM, and miRNA). Color scale indicates presence or absence of significant annotation based on hypergeometric testing. **(B, C)** share the same color scale, indicating average expression (Z-score normalization) per disease group-cell type (rows). Gene markers are in the columns, classified in three groups according to their shared expression patterns across cell types: “Tri-lineage” denotes genes upregulated in monocytes, *in vitro* moDCs, and tolDCs. “Monocyte (UP)” refers to genes consistently upregulated in monocytes, while “moDCs (DOWN)” indicates genes consistently downregulated in *in vitro* moDCs. **(B)** SLE-associated genes and **(C)** Differentially expressed ISGs (DEISG); dot size reflects the percentage of samples expressing each marker. **(D)** Heatmap of biological processes associated with the DEGs. Processes were classified according to Reactome (REAC) parent and specific categories, and Gene Ontology Biological Process (GO : BP) parent terms. REAC parent processes include Immune system, Synthesis of phosphatidylinositol (PI), Cell division, Lipid metabolism and Transport of small molecules. Color scale represents padj (FDR–BH correction), with a maximum threshold of padj < 0.05. SLE, Systemic Lupus Erythematosus; Ctrl, healthy control; mo, monocytes; moDCs, *in vitro* monocyte-derived dendritic cells; and tolDCs, tolerogenic dendritic cells; DEGs, differential gene expression; padj, adjusted p-value; LFC, log_2_FoldChange; FDR, false discovery rate; BH, Benjamini–Hochberg procedure.

To understand the general differential expression signature of SLE in monocytes, *in vitro* moDCs and tolDCs, we generated transcriptomic data for each cell type (**Figure 2A**). PCA of normalized expression confirmed the separation between cell types and the absence of batch effects (variance explained by PC1: 62.23%, PC2: 9.33%) (**Supplementary Figure 2**). Regarding disease activity estimated with SLEDAI, PCA (**Supplementary Figure 2C**) did not reveal a clear separation of samples according to SLE disease activity, suggesting it was not the primary driver of transcriptomic variability in our dataset. We performed differential expression analysis between SLE and controls for each cell type. In total, we identified 94 differentially expressed genes (DEGs) across all comparisons (**Supplementary Table 5**). These comprised: 37 DEGs in monocytes, all of them upregulated in SLE (**Figure 2C**); 44 in *in vitro* moDCs (4 upregulated and 40 downregulated) (**Figure 2D**); and 16 in tolDCs (4 upregulated and 12 downregulated) (**Figure 2E**). Their expression patterns are shown in **Figures 3A**, highlighting cell type-specific expressions.

Established SLE transcriptional signatures have been widely reported in prior studies. Hence, we integrated a reference list of SLE-associated genes (**Supplementary Table 6**) used to assess the disease relevance of our findings. We observed a significant enrichment of SLE-associated genes among the set of DEGs (hypergeometric test, p-value = 1.144 × 10^-11^; Fisher’s exact test, p-value = 1.144 × 10^-11^) (**Figure 3B**; **Supplementary Table 5**). Specifically, monocytes from SLE patients showed elevated expression of *IFI27*, *CMPK2, EIF2AK2, IFI44L, IFI6, IFIT1, IFIT3, ISG15, OAS1, OAS2, RSAD2* and *USP18*, all of them well−established SLE-associated genes (**Figure 3B**). In contrast, among *in vitro* moDCs we found downregulation of one gene previously associated with SLE: *LBH* (**Figure 3B**); and no SLE-associated genes were significant for tolDCs. These findings point to cell type-specific transcriptional shifts that may alter DCs signaling and immune regulation in SLE.

Interferon-stimulated genes (ISGs) are central drivers of monocyte and dendritic cell activation, and their persistent upregulation is a defining feature of SLE (57). Hence, we set out to identify ISGs within our DEG using a compiled list (17) (**Figure 3C**, **Supplementary Table 7**). ISGs were overrepresented among DEGs (hypergeometric test, p-value = 2.48 × 10^-31^; Fisher’s exact test, p-value< 2.2 × 10^-16^). Among all ISGs, *IFI27* was the only gene up−regulated across all three cell types in SLE (denoted as Tri-lineage in **Figures 3B, C**), underscoring its potential relevance in SLE pathophysiology (58–60).

Specifically, monocytes from SLE patients exhibited 30 differentially expressed ISGs (DEISGs) (**Figure 3C**; **Supplementary Table 7**). Among these, differences in *IFI27*, *IFI6*, *IFI44L* and *USP18* expression are consistent with previous reports, supporting their strong potential as biomarkers to distinguish SLE from controls; in addition, the marked enrichment of these ISGs in monocytes highlights a potentially central role of monocytes in sustaining lupus−associated transcriptional programs (59–65). In contrast, among *in vitro* moDCs we found two ISGs that had differential gene expression between SLE and healthy controls (*AUTS2* and *IL2RB/CD122*), and no ISGs were significant for tolDCs (**Figure 3C**). Replicating results previously showing no significant differences found between SLE patients and healthy controls (20).

ISGs expression clearly separates SLE patients from healthy controls. Accordingly, we assessed the relationship between ISGs levels and SLEDAI by cell type using Spearman correlation; however, no significant association was detected. (**Supplementary Table 8**). This likely reflects the influence of immunomodulatory treatments and the dampening of *in vivo* inflammatory signals during *in vitro* differentiation.

Pathway enrichment analysis of DEGs revealed immune−related Reactome (REAC) pathways (**Figure 3D**; **Supplementary Table 7**). Among monocyte DEGs, the most frequent parent process (BP) was immune system related processes, among which specific process (REAC) included: interferon alpha/beta signaling emerged as the most significantly enriched pathway (*IFI6, IFIT3, IFITM3, RSAD2, XAF1*), followed by ISG15 antiviral mechanism (*IFIT1, MX1, MX2, USP18*), and OAS antiviral response (*OAS1, OAS2, OAS3, OASL*) and maturation of nucleoprotein (*PARP14* and *PARP9*) (See full list of terms in **Supplementary Figure 4**; **Supplementary Table 9**). Interferon signaling plays a decisive role in reshaping monocyte transcriptional programs in SLE, a process that likely underpins the systemic inflammatory and autoimmune manifestations characteristic of the disease (17, 58, 64). For *in vitro* moDCs, *IL2RB* was associated with the immune system, whereas *AUTS2* was not found to be associated with any specific process. Regarding tolDCs, no ISGs or SLE-associated genes were observed.

To investigate the regulatory layer behind these DEGs, we assessed whether regulon activity differed between SLE and control samples within each DCs type, using AUC-based regulon scores and the statistical framework described above (See Methods). After multiple-testing correction (LFC > 0 and padj< 0.05), no regulons reached statistical significance in any of the cell types analyzed. This suggests that, at the level for these specific cell types, transcriptional regulatory changes between SLE and controls are subtle and do not manifest as robust, global shifts in regulon activity, confirming previous findings (20).

### Immune and metabolic remodeling during moDCs and tolDCs *in vitro* differentiation in SLE

**Figure 4:**
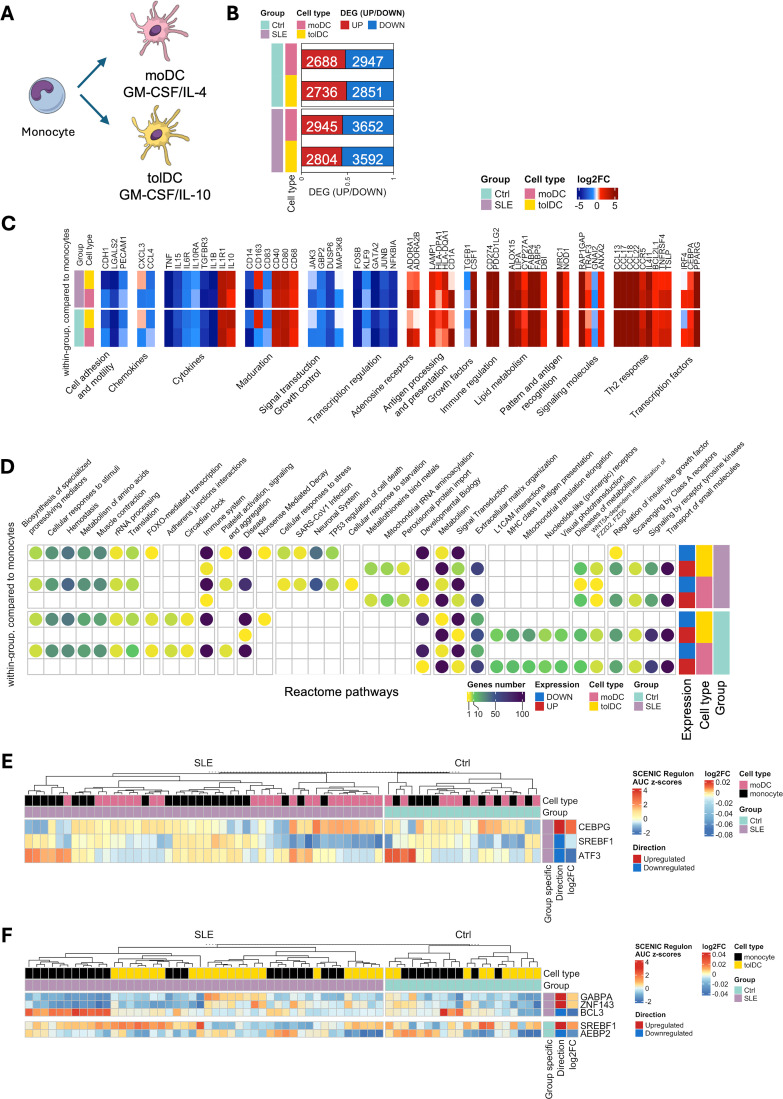
Monocyte differentiation to moDCs and tolDCs. **(A)** Schematic representation of monocyte *in vitro* differentiation into DCs (moDCs and tolDCs). **(B)** Number of differentially expressed genes (DEGs) in DCs of both SLE and controls, using monocytes as reference. **(C)** Genes previously reported as up− or downregulated during moDCs *in vitro* differentiation compared with monocytes, showing the expected transcriptional changes in our dataset. **(D)** Biological processes enriched among DEGs. **(E)** Exclusive regulons associated with monocyte *in vitro* differentiation into *in vitro* moDCs. **(F)**. Exclusive regulons associated with monocyte differentiation into tolDCs. Significance thresholds were set at absolute LFC ≥ 0.5 and padj< 0.05. SLE, Systemic Lupus Erythematosus; Ctrl, healthy control; mo, monocytes; moDCs, *in vitro* monocyte-derived dendritic cells; and tolDCs, tolerogenic dendritic cells; DEGs, differential gene expression; padj, adjusted p-value; LFC, log_2_FoldChange; FDR, false discovery rate; BH, Benjamini–Hochberg procedure.

Our initial analyses revealed that most transcriptional variation was attributable to cell type identity, with minimal differences between SLE and controls; this was also reflected in the regulon profiles in which the major source of variation corresponds to cell type instead of condition (**Supplementary Figure 5**), and tolDCs showed no disease-specific signals. Consequently, we focused on comparing the differentiation trajectories themselves, assessing how the monocyte to moDCs/tolDCs *in vitro* differentiation changed between SLE and healthy individuals (**Figure 4A**). In total, we found over 8,114 unique DGEs whose expression levels changed significantly across these comparisons (4427 upregulated and 3752 downregulated). We found: 2,688 up and 2,947 downregulated DEGs in monocytes vs *in vitro* moDCs from controls; 2,736 up and 2,851 downregulated DEGs in monocytes vs tolDCs from controls; 2,945 up and 3,652 downregulated DEGs in monocytes vs *in vitro* moDCs from SLE; and 2,804 up and 3,591 downregulated DEGs in monocytes vs tolDCs from SLE (**Figure 4B**; **Supplementary Table 10**).

Gene expression studies of monocyte differentiation into *in vitro* moDCs have consistently identified characteristic transcriptional changes that define a differentiation signature. A hallmark of this process is the downregulation of classical monocyte markers, including *CD14* and *CD163* (12, 45). In our analysis, we confirmed that the *CD14* gene was repressed in both *in vitro* moDCs and tolDCs, in agreement with earlier studies (12). However, *CD163* displayed a distinct pattern: while downregulated in *in vitro* moDCs as expected, it was upregulated in both tolDCs from SLE and controls (**Figure 4C**; **Supplementary Table 10**). Compared what has been reported, we also observed overexpression in tolDCs differentiations (both SLE and controls) of genes related to DCs maturation including *CD40, CD80* and *CD68*, and with the production of anti-inflammatory cytokines, such as *IL-10* and *IL1R1* (**Figure 4C**; **Supplementary Table 11**) which are required for tolerance induction (59). Furthermore, *in vitro* moDCs and tolDCs from both SLE patients and controls showed significant downregulation of *CD83*, a marker associated with DCs maturation (60, 62, 65) (**Figure 4C**).

Consistent with previous reports, we found that *in vitro* moDCs exhibited upregulation of genes associated with recognition and antigen uptake (*MRC1* and *NOD1*), antigen processing and presentation (*LAMP1, HLA-DPA1, HLA-DQA2* and *CD1a*), growth factors (*CSF1*), cytokines and their receptors (*IL-10* and *IL1R1*), Th2 response (*CCL13/MCP-4, CCL17/TARC, CCL18/PARC, CCL22/MDC, CCR5, IL4I1, BCL2L1, TNFRSF4* and *TSLP*), lipid metabolism (*ALOX15, LIPA, CYP27A1, FABP4* and *FABP5*), adenosine receptors (*ADORA1* and *ADORA2B*), signaling molecules (*RAP1GAP, TRAF3*, and *ANXA2*), and transcription factors (*IRF4, CEBPA/C/EBP–α*, and *PPARG/PPAR-γ*) (**Figure 4C**). In contrast, we observed downregulation of genes associated with cell adhesion and motility (E-cadherin/*CDH1*, galectin-2/*LGALS2* and *PECAM1*/*CD31*), chemokines (*CXCL3*/MIP-2β and *CCL4*/MIP-1β), cytokines and cytokine receptors tumor necrosis factor (TNF)-α (*TNF*, *IL-15*, *IL-6R*, *IL10RA* and *TGFBR3*), signal transduction/growth control (*JAK3*, *GBP2*, *DUSP6*, and *MAP3K8*), and transcriptional regulators (*FOSB*, *KLF9*, *GATA2*, *JUNB* and *NFKBIA*) (**Figure 4C**). Notably, in contrast to earlier studies, we also observed downregulation of *TGFB1, GNAI2*, and *IL-1β*, which had previously been reported as upregulated in *in vitro* moDCs (12, 63).

A similar expression pattern was observed between differentiation trajectories in *in vitro* moDCs and tolDCs, with two major exceptions: *MAP3K8* was not differentially expressed in tolDCs comparisons, whereas *CXCL3* was consistently upregulated in tolDCs, while downregulated in *in vitro* moDCs (**Figure 4C**). Specifically, in tolDCS, the overall expression pattern was maintained, except for *IRF4*, which was downregulated only in controls showing no significant difference in SLE (**Figure 4C**).

Pathway enrichment analysis of DEGs revealed distinct patterns associated with monocyte to moDCs *in vitro* differentiation for both SLE and controls (**Figure 4D**). In controls, we observed downregulation of pathways including: FOXO-mediated transcription, adherents junctions’ interactions, extracellular matrix organization and circadian clock regulation (**Figure 4D**). Conversely, upregulation of pathways associated with *L1CAM* interactions, MHC class II antigen presentation, mitochondrial translation elongation, nucleotide-like purinergic receptors and visual phototransduction was observed. For only SLE, several pathways were downregulated, including cellular response to stress, SARS-CoV-1 infection, neuronal system, *TP53* regulates transcription of cell death gene and cellular response to starvation. In contrast, we found upregulation of metallothionein bind metals, mitochondrial tRNA aminoacylation, and peroxisomal protein import.

Pathways involved in the monocyte to tolDCs differentiation for controls included the upregulation of *L1CAM* interactions, MHC class II antigen presentations, mitochondrial translation elongation, nucleotide-like purinergic receptors, visual phototransduction, and disease. In contrast, downregulated pathways among tolDCs controls included: extracellular matrix organization. SLE tolDCS showed upregulation of pathways associated to metallothionein binds metals, mitochondrial tRNA aminoacylation, and peroxisomal protein import, with downregulation of regulation of insulin-like growth factor (*IGF*), cellular response to stress, SARS-CoV-1 infection, neuronal system and TP53 regulates transcription of cell death gene (**Figure 4D**).

To understand the regulatory mechanisms particularly driving the differentiation process to DCs phenotypes, we built gene regulatory networks (see Methods). We identified 15 upregulated and 24 downregulated regulons (absolute log_2_FC > 0 and adjusted p-value< 0.05) upon *in vitro* differentiation to moDCs (**Figure 3E**), that were common between SLE and controls. We identified one upregulated (*CEBPG*) and two downregulated (*ATF3* and *SREBF1*) SLE-specific regulons during *in vitro* differentiation into moDCs. We didn’t find any up or downregulated regulons that were unique to the control samples in this same process (**Figure 4E**). In the differentiation towards tolDCs, we identified 12 upregulated and 27 downregulated regulons in common between SLE and healthy controls (**Figure 4F**). When inspecting SLE-specific regulons, we found two upregulated (*GABPA* and *ZNF143*) and one downregulated (*BCL3*). In addition, we found one control-specific upregulated regulon (*SREBF1*) and one downregulated regulon (*AEBP2*).

To further examine the relevance of these regulons in SLE, we analyzed the target genes of regulon TFs that were uniquely associated with SLE during differentiation into DCs phenotypes. For this, we subset those that were also found to be differentially expressed. We found that the downregulated regulon *BCL3* in the tolDCs differentiation process was composed mostly of downregulated genes (LFC< 1 in DE), while the upregulated regulon targets had a mixed regulation direction (**Supplementary Figure 6**). In addition, we note that the downregulated regulon upon *in vitro* moDCs differentiation, *SREBF1*, regulates *IRF35* and *STAT1*, which have been found as part of the SLE-Related Monocyte signature (SLERRA signature) (54)*. STAT1* was found in our DE analysis as downregulated with LFC = -1.63.

We next conducted gene enrichment analysis of the target genes of the significant regulons involved in the differentiation of both dendritic cell phenotypes. We specifically focused on target genes that were also differentially expressed during the corresponding phenotype-specific differentiation. We found that in *in vitro* moDCs, the term “*FGFR1* mutant receptor activation” was enriched for genes targets of the upregulated regulons. In contrast, the targets for regulons upregulated in tolDCs were enriched in terms related to response to vasopressin. More interestingly, we found multiple extracellular signal pathways, such as *FLT3*, growth hormone receptor and interleukin-7 signaling to be associated with the gene target *SOCS2* (DE LFC = -1.14), of the downregulated regulon *BCL3* in tolDCs (**Supplementary Figure 7**).

**Figure 5:**
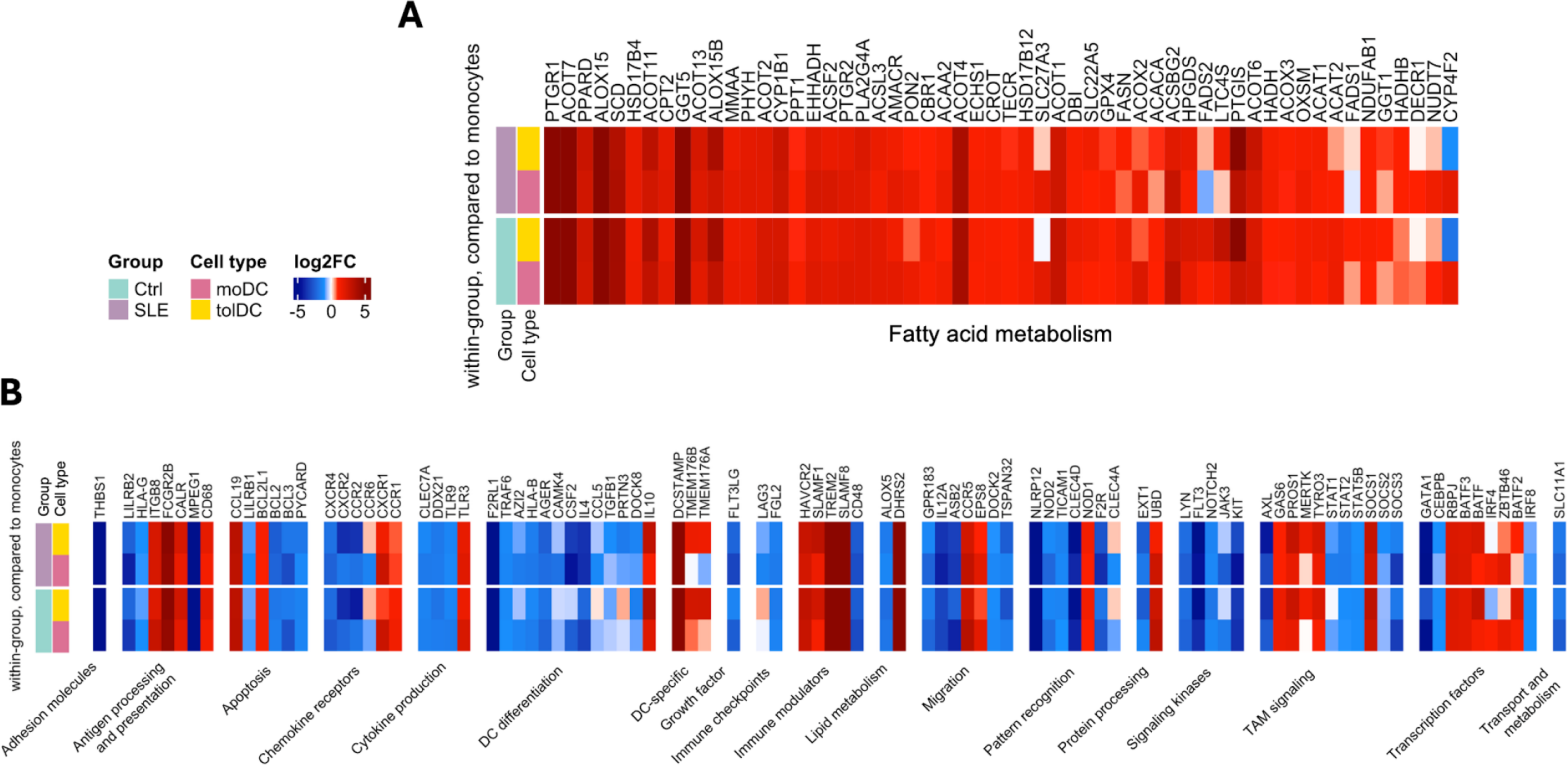
Differentially expressed genes (DEGs) between DCs and monocytes from SLE patients and healthy controls, related to DCs differentiation. **(A)** Genes associated with fatty acid metabolism, reflecting metabolic reprogramming during the transition from monocytes to DCs. **(B)** Genes linked to canonical DCs differentiation pathways. Significance thresholds were set at absolute LFC ≥ 0.5 and padj< 0.05. SLE, Systemic Lupus Erythematosus; Ctrl, healthy control; mo, monocytes; moDCs, *in vitro* monocyte-derived DCs; and tolDCs, tolerogenic DCs; DEGs, differential gene expression; padj, adjusted p-value; LFC, log_2_FoldChange.

Lipid metabolism and associated mechanisms have been reported to be involved in the pathogenesis and progression of SLE (17). Hence, we explored the contribution of previously identified DEGs (66) associated with lipid metabolism to the *in vitro* differentiation of monocytes into moDCs and tolDCs. We detected 56 differentially expressed genes (DEGs) in *in vitro* moDCs and tolDCs from SLE patients compared with controls, which are associated with β-oxidation, fatty acid synthesis, and desaturation pathways, including members of the ACOT family, *ACSL3*, *FASN*, *ACACA*, *ACOX2/3*, *HADH*, and *HADHB* (**Figure 5A**).

In the moDCs *in vitro* differentiation trajectory (**Figure 5A**), we found that *FADS2* was downregulated in SLE patients but upregulated in all other comparisons, suggesting impaired desaturation of polyunsaturated fatty acids in SLE (66). We also identified DEGs related to peroxisome proliferator−activated receptors (PPARs), transcription factors that regulate lipid metabolism and immune function, which have been reported to be significantly upregulated in *in vitro* moDCs generated using GM−CSF and IL−4 (67, 68). Regarding tolDCs, differentiation and lipid metabolism, we observed downregulation of *CYP4F2* on both controls and SLE, consistent with reduced production of lipid−derived inflammatory mediators in tolDCs; previous studies have linked *CYP4F2* lipid metabolism function with immune regulation (69). We also identified the downregulation of *SLC27A3* in tolDCs from controls and SLE (**Figure 5A**). Together, these findings underscore the importance of lipid metabolic reprogramming in shaping DCs differentiation and function (**Figure 5A**).

To further characterize differentiation trajectories, we analyzed genes annotated in the Gene Ontology Biological Process (GO: BP) category for DCs differentiation. This exploration revealed additional transcriptional programs that define the functional identities of *in vitro* moDCs and tolDCs (67). In *in vitro* moDCs, *CCR6* was downregulated in both SLE and control samples, unlike in tolDCs, where its expression was preserved/consistent with a role for *CCR6* in supporting the migratory and regulatory features of the tolerogenic phenotype. Conversely, *CLEC4A* exhibited divergent regulation, being upregulated in *in vitro* moDCs from both SLE patients and healthy controls (**Figure 5B**). In tolDCs, *MERTK* was selectively upregulated in tolDCs from both SLE and controls, whereas *STAT1* showed downregulation only in SLE.

Genes that displayed differential expression in only one of the differentiation trajectories (monocytes to *in vitro* moDCs or monocytes to tolDCs) within either SLE or control samples were considered trajectory specific. Several genes showed exclusive or divergent patterns across groups. For example, *CCL5* and *PRTN3* were upregulated during monocyte-to-moDCs *in vitro* differentiation in healthy controls but were downregulated in all other trajectories, underscoring their context-dependent roles. *LAG3* also exhibited a disease-linked pattern, being downregulated in both SLE *in vitro* moDCs and tolDCs while remaining unchanged in controls (**Figure 5B**).

**Figure 6:**
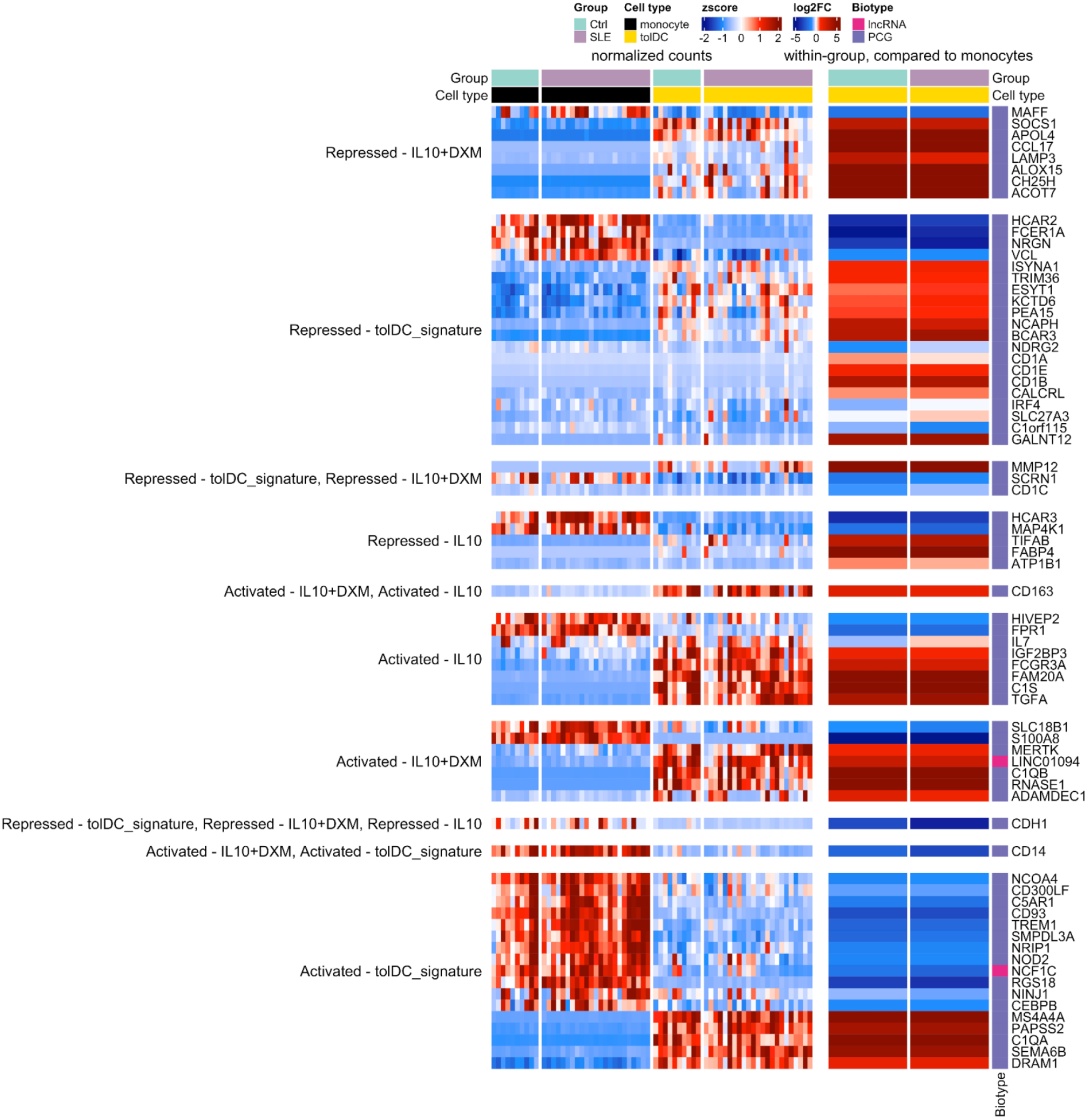
Transcriptional signatures associated with tolDCs obtained by SLE vs controls comparison. Heatmaps showing 71 DEGs associated with tolDCs transcriptional signatures (32 downregulated and 41 upregulated) comparing monocytes vs DCs in SLE and controls. Rows represent genes and columns represent samples, colored according to cell types (monocytes, *in vitro* moDCs and tolDCs) and SLE vs Ctrl. Left panel: heatmap displays normalized gene expression values (z−score). Right panel: heatmap shows LFC values relative to monocytes. Significance thresholds were set at absolute LFC ≥ 0.5 and padj< 0.05. Biotypes are color−coded: purple for protein−coding genes (PCG) and pink for long non−coding RNAs (lncRNAs). SLE, Systemic Lupus Erythematosus; Ctrl, healthy control; and tolDCs, tolerogenic dendritic cells; LFC, log_2_FoldChange.

TolDCs can be generated through various stimuli, such as IL-10 or dexamethasone, each producing a distinct transcriptional profile (48). To assess how our differentiation conditions influenced the resulting transcriptomes, we compared the monocyte to tolDCs trajectory DEGs in both SLE and control samples with these previously reported stimulus-specific profiles, as well as with the general tolDCs signature described in the same study (48). In this analysis, we identified 71 DEGs associated with the tolDCs signature, of which 32 were previously identified as repressed and 41 as activated in tolDCs (48). This analysis of tolDCs signatures revealed both concordant and divergent gene expression patterns with our dataset. Several genes matched previously reported activation (13 genes) and repression profiles, while others showed opposite regulation (27 genes) (**Supplementary Table 12**). Interestingly, *IL-7* displayed a disease-specific regulatory signature in SLE, being overexpressed in tolDCs from SLE patients but downregulated in tolDCs from controls, highlighting a potential disease-specific modulation of the tolerogenic signature. When focusing specifically on the IL-10 derived tolDCs we found that 12 of the top 20 reported by Robertson et al. (48), were also differentially expressed in our tolDCs, supporting that our *in vitro* protocol recapitulates for the most key features of IL-10 driven tolerogenic programming (**Figure 6**).

### Interaction of SLE on monocyte to *in vitro* moDCs and tolDCs transcriptional programs

**Figure 7:**
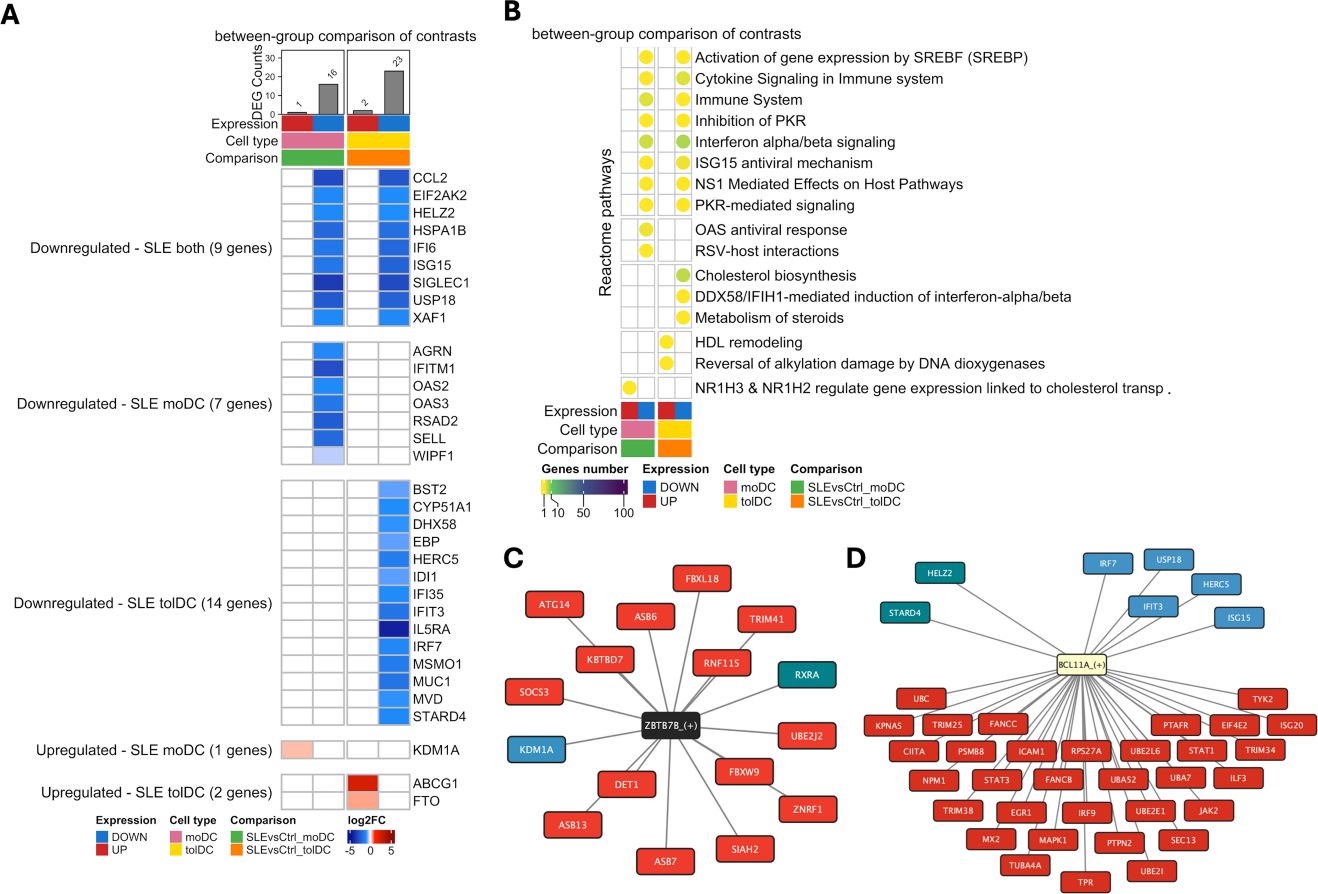
Monocyte−derived dendritic cells (*in vitro* moDCs and tolDCs) and associated transcriptional programs obtained by SLE vs. Ctrl comparison. **(A)** Heatmap of differentially expressed genes (DEGs) that are shared or unique to *in vitro* moDCs and tolDCs, using monocytes as baseline. **(B)** Significant biological processes implicated in SLE patients within these comparisons. **(C)** Network of *ZBTB7B* highlighted by pathway. Red: Targets associated with the global and biggest pathway (Class I MHC mediated antigen processing & presentation/Interferon Signaling), Blue: *KDM1A*/*LSD1*, target from DE results associated with cholesterol transport and efflux, Green: *RXRA*, target associated with lipid metabolism in which *KDM1A*/*LSD1* is involved. **(D)** Network of *BCL11A* targets highlighted by origin. Red: targets of *BCL11A* not present in DE results and associated with interferon signaling; Blue: *BCL11A* targets present in DE results and associated with interferon signaling; Green: BCL11A targets associated present in DE analysis not associated with interferon signaling.

Our previous differentiation analysis provided a useful baseline for understanding how monocytes progress toward *in vitro* moDCs and tolDCs states in SLE and controls. However, this approach does not reveal whether the differentiation process unfolds differently in patients compared with controls. We therefore carried out a second analysis focused explicitly on identifying disease-dependent modifications to the differentiation trajectory, integrating SLE as an interaction factor (see Methods). We identified 33 DEGs exclusively in SLE (3 upregulated and 30 downregulated) (**Supplementary Figure 8**). Among these, 9 downregulated genes (*CCL2, EIF2AK2, HELZ2, HSPA1B, IFI6, ISG15, SIGLEC1, USP18*, and *XAF1*) were shared between *in vitro* moDCs and tolDCs (**Figure 7A**), mainly involved in interferon signaling, antiviral response, and immune regulation (**Figure 7B**). Seven downregulated genes specific to *in vitro* moDCs (*AGRN, IFITM1, OAS2, OAS3, RSAD2, SELL*, and *WIPF1*) (**Figure 7A**), were linked to cell adhesion, viral restriction, and innate immune activation (**Figure 6B**). Fourteen downregulated genes specific to tolDCs (*BST2, CYP51A1, DHX58, EBP, HERC5, IDI1, IFI35, IFIT3, IRF7, MSMO1, MUC1, MVD*, and *STARD4*) (**Figure 6A**), were associated with cholesterol biosynthesis, interferon-stimulated pathways, and transcriptional regulation of immune responses (**Figure 7B**). In contrast, two genes upregulated in tolDCs (*ABCG1* and *FTO*) were related to lipid transport and metabolic regulation, while *KDM1A/LSD1* upregulated in *in vitro* moDCs was linked to epigenetic modulation of transcription (**Figures 7A, B**) and were found having a higher activity in SLE during differentiation (**Supplementary Figure 9**). These findings highlight that the transcriptional changes observed in DCs are strongly influenced by disease status, as reflected by the differential expression of 33 genes (mean |LFC| = 1.72, range 0.34–4.29, adj. p< 0.05), underscoring the weight of SLE in shaping the regulatory programs of monocyte-derived DCs.

To further investigate the transcriptional activity associated with this gene set, we constructed a targeted Gene Regulatory Network. Given that we did not find differential regulons between the SLE and control groups when introducing SLE interaction (**Supplementary Figures 10**, **11**) and the PCA did not reveal global changes in regulon activity between SLE and controls (**Supplementary Figure 12**). We decided to select regulons whose predicted targets overlapped with DEGs shared or unique to *in vitro* moDCs and tolDCs (**Supplementary Table 13**) and focused on those showing the strongest differential activity in tolDCs. *ZBTB7B* showed the largest regulon-level activity difference between SLE and control samples in tolDCs (**Supplementary Figure 12**) and was borderline enriched in the interaction analysis (70). However, only one predicted target of this regulon, *KDM1A* (*LSD1*), was identified as differentially expressed in *in vitro* moDCs (**Figure 7C**). With the aim of exploring this regulon in greater detail, we also decided to examine an extended set of targets that are part of the larger pathway in which *KDM1A* is involved, and we identified 14 additional genes associated with antigen processing & presentation and 1 more gene associated with lipid metabolism (**Figure 7C****).**

*BCL11A* emerged as the most strongly downregulated regulon in tolDCs from SLE samples (**Supplementary Figure 13**). Seven predicted *BCL11A* target genes were differentially expressed, five of which, including *USP18*, *ISG15*, *IRF7*, *IFIT3*, and *HERC5*, are annotated as interferon-related genes (**Figure 7D**). Among these, *USP18* and *ISG15* were also differentially expressed in monocytes and remained dysregulated in tolDCs. Additionally, we found 33 genes associated with interferon signaling that were not present in our DE results and 2 genes present in our DE results but not associated with interferon signaling (**Figure 7D**).

Finally, we asked whether interferon-driven transcriptional programs present at baseline in monocytes are carried forward during differentiation into DCs. Focusing on genes shared between the monocyte SLE vs. control comparison and the interaction effect, we found that 18 of the 37 monocyte DEGs overlapped, including fourteen interferon-related transcription factors and their targets. These results indicate that type I interferon signaling imprinted in SLE monocytes is propagated into subsequent DCs differentiation (**Supplementary Table 14**).

## Discussion

The breakdown of immune tolerance is a central mechanism of SLE pathogenesis, promoting the activation of autoreactive cells and the production of autoantibodies. Due to their pivotal role at the interface of innate and adaptive immunity, DCs are key in modulating the immune response, and their dysregulation contributes to both the breakdown of immune tolerance and chronic inflammation in SLE. A deeper understanding of the mechanisms underlying DCs functional phenotypes in people with SLE could provide significant insights for therapeutic approaches.

We compared the transcriptomic profiles of monocyte-derived dendritic cells from Mexican women with and without SLE and found that ISGs are strong molecular markers across cell types, highlighting the central role of interferon signaling in SLE pathophysiology (71). We identified numerous ISGs differentially expressed across all three cell types, enriched in interferon-driven pathways. Monocytes showed the strongest ISG upregulation, including *ISG15*, a negative regulator of type I IFN previously reported to be elevated in SLE monocytes (71). This finding is consistent with the well-established role of type I IFN pathways in the pathogenesis of SLE, and it was related to the previously reported SLERRA signature (17). Suggesting that monocytes may contribute importantly to the SLE-associated interferon transcriptional profile, although the signature remains in derived DCs.

In addition to the enrichment of ISGs in monocytes, we identified several SLE biomarkers previously reported to be related to hub ISGs, including *IFI27, CMPK2, EIF2AK2, IFI44L, IFI6, IFIT1, ISG15, OAS1, OAS2, RSAD2, USP18*, and *LBH* (64, 72). These findings align with previous studies showing that interferon signaling is central to SLE pathogenesis, with ISG overexpression (“interferon signature”) present in up to 80% of patients and associated with disease activity (72–74). Among these, we detected increased expression of *IFI44L, IFI27, USP18* and *IFI6*, a four-gene interferon signature with strong diagnostic performance, highlighting their central role in interferon-driven transcription and immune dysregulation in SLE (75).

*USP18* showed the third highest fold change (LFC = 3.123, paj = 1.371e-05), highlighting its potential relevance to SLE pathophysiology given its role as a negative regulator of interferon signaling (76). *IFI27* was consistently overexpressed across all three cell types, underscoring its key role in immune modulation, apoptosis, and antiviral responses (17, 64). Notably, interferon-inducible protein 44-like (*IFI44L*), promotes moDC maturation by upregulating costimulatory molecules (CD40, CD80, CD83, and CD86) and driving Th1/Th17 polarization, potentially contributing to loss of tolerance and chronic inflammation in SLE (77). Together, the persistent overexpression and functional roles of these ISGs highlight their potential as therapeutic targets to modulate IFN signaling in SLE.

Beyond the interferon-driven transcriptional signature, tolDCs from SLE patients exhibited changes enriched in metabolic and proliferative pathways. Genes involved in cholesterol and lipid metabolism (e.g. *ACAT2, MSMO1*, and *SMPD3*), lipid/cholesterol efflux (e.g. *ABCA1, ABCG1*), and regulators of the cell cycle (e.g. *CDCA5, CENPM*) were exclusively dysregulated in SLE tolDCs. These findings align with growing evidence regarding the role of lipid metabolism in the regulation of DCs responses; efficient cholesterol efflux and lipid metabolism is key to maintaining immune tolerance in DCs: impaired mechanisms promote accumulation of intracellular lipids, activate inflammasome and secrete pro-inflammatory cytokines, promoting autoreactive T and B cell responses and contributing to chronic inflammation and autoimmune responses (78–81). This dysregulation tolDCs from SLE, supports a model in which metabolic reprogramming contributes to DCs dysfunction, and highlights lipid homeostasis inducing strategies as potential targets to restore immune tolerance in SLE.

In this study, we observed limited differentially expressed genes in monocytes, *in vitro* moDCs, and tolDCs when analyzed using direct SLE versus controls comparisons. Disease associated transcriptional signals present *in vivo*, such as inflammatory cytokines, immune complexes, and interferon signaling may be partially attenuated during *in vitro* culture, thereby masking baseline differences between patient-derived and control-derived cells (82, 83). Importantly, variability in disease activity within the SLE cohort (43.5% active and 56.5% inactive) likely contributed to the limited detection of disease-associated differences in direct case–control comparisons (71, 83, 84). This observation is consistent with the well-recognized clinical and molecular heterogeneity of SLE, as well as the high plasticity of myeloid cells and the partial normalization of environmental signals under controlled *in vitro* differentiation conditions (85).

To further investigate differences between tolDCs and *in vitro* moDCs, we analyzed the differentiation trajectories from monocytes to each DCs subtype and compared the resulting gene expression changes between SLE and controls. As expected, differentiation induced strong remodeling, with over 8,000 DEGs, including downregulation of monocyte markers (e.g. *CD14*), upregulation of DCs maturation and antigen presentation markers (e.g. *CD40, CD80, CD68, HLA-DPA1*, and *HLA-DQA1*), anti-inflammatory mediators (e.g. *IL10, IL1R1*), and the downregulation of other classical maturation markers such as *CD83* (16). These transcriptional changes validate that our *in vitro* differentiation protocol successfully induces DCs features, providing a robust model to explore disease-driven alterations.

Beyond these expected changes, we identified shared transcriptional profiles of DCs differentiation in both SLE and controls (**Figure 4**). In *in vitro* moDCs, downregulation of *CXCL3, JAK3*, and *MAP3K8* suggests attenuation of early inflammatory signaling, while upregulation of *ADORA1/2B, CD1A, LIPA, TRAF3, CCR5, TNFRSF4, TSLP*, and *IRF4* reflects activation of pathways that support DCs maturation, lipid antigen handling, and purinergic regulation (79, 86–89). Notably, moDCs from SLE patients exhibited lower *IL10RA* expression, which may impair IL-10–mediated tolerogenic signaling via the IL10RA–JAK1–STAT3 axis, despite elevated systemic IL-10 levels in SLE (90, 91). In contrast, tolDCs from controls exhibited lower *IRF4* expression, a key driver of DCs maturation and antigen-presenting function; its reduced expression in controls is consistent with a physiologically restrained, steady-state maturation program (92–94). This higher *IRF4* gene expression in SLE *in vitro* moDCs (LFC = 1.44, padj< 0.05) likely reflects chronic *in vivo* inflammatory priming, consistent with the pre-activated status previously described in SLE myeloid cells (95, 96). Together, the diminished *IL10RA* (LFC = -0.73) and dysregulated *IRF4* (LFC = 1.44) in SLE support the presence of cell-intrinsic defects in peripheral tolerance that may predispose antigen-presenting cells toward heightened activation.

Regarding tolDCs, both SLE and controls exhibited a transcriptional profile that resembles the human IL−10 producing DCs (DC-10) subset, characterized by high *IL-10* (Ctrl LFC = 3.60, SLE LFC = 3.14) and *CD163* expression (Ctrl LFC = 2.07, SLE LFC = 1.74). This is consistent with the IL-10 driven differentiation conditions we used to generate tolDCs, as CD163 is a cytokine-regulated marker associated with specific tolDCs subsets, including DC-10. Accordingly, CD163 upregulation in tolDCs likely reflects the acquisition of a tolerogenic transcriptional program shaped by IL-10 exposure (90, 97, 98). Interestingly, despite this tolerogenic signature, we observed an unexpected upregulation of *HLA-DPA1* (Ctrl LFC = 0.88, SLE LFC = 1.16) and *HLA-DQA* (Ctrl LFC = 1.89, SLE LFC = 3.00). This pattern may reflect the preservation of antigen-presenting capacity which, in the context of high IL-10 (Ctrl LFC = 3.60, SLE LFC = 3.14) and reduced co-stimulation (CD80/CD40) could favor the presentation of self-antigens to promote T cell anergy or regulatory T cell induction (99, 100). Accordingly, the phenotype observed in our study, characterized by CD80 upregulation and CD83 downregulation, likely reflects incomplete terminal maturation of both immature moDCs, which were not exposed to maturation stimuli, and IL-10 driven tolDCs, consistent with established tolDCs differentiation protocols (97, 101, 102). IL-4, a key component of our differentiation protocol, reinforces this phenotype by inducing sustained PPAR-γ activity, which limits full DC maturation and pro-inflammatory T-cell priming (103). TolDCs exhibited a coordinated transcriptional program with elevated HLA class II expression alongside IL-10/PPAR-γ–mediated immunoregulatory signaling, consistent with antigen presentation in an anti-inflammatory context. Notably, tolDCs from SLE donors showed greater upregulation of *ADORA2B*, a purinergic receptor that promotes tolerogenic reprogramming by enhancing *IL-10* production and suppressing pro-inflammatory cytokines (104, 105). Although both SLE and controls exhibited a DC-10-like transcriptional profile, the differences found between SLE, and controls suggest different molecular pathways towards tolerogenic programming.

We next examined molecular pathways underlying the differences observed in the trajectory analysis comparing monocytes to *in vitro* moDCs and tolDCs in SLE and controls (**Figure 5**). This analysis revealed pronounced alterations in lipid metabolism, with several DEGs involved in lipid metabolism alongside increased expression of lipid regulatory transcription factors such as PPARγ and PPARβ/δ (106, 107). We observed reduced expression of *FADS2* and *FADS1* in *in vitro* moDCs from SLE, key enzymes for long-chain polyunsaturated fatty acids and pro-resolving mediator synthesis, consistent with impaired pro-resolving lipid mediator production in SLE (108, 109). Several lipogenic genes observed in our dataset (e.g., FASN, ACACA) have been reported in other studies to be regulated by the aryl hydrocarbon receptor *(AhR)* (110–112). Although AhR itself was not detected as a regulon or DEG in our analysis, prior work has implicated AhR downstream of IL-10 signaling in DCs, where it contributes to tolerogenic programming and metabolic regulation (111, 113, 114). These observations provide contextual support for the link between lipid metabolism and tolerogenic differentiation, but direct involvement of AhR in our system remains to be determined.

Regarding other molecular pathways, *in vitro* moDCs from controls showed upregulation of *CCL5*, indicating enhanced chemotactic signaling and T-cell recruitment consistent with DCs maturation (115). Additionally, downregulation of *TMEM176B* and *TMEM176A*, genes previously associated with immature or regulatory states of DCs, supports a shift toward a more immunogenic phenotype (116). These findings suggest that control moDCs differentiate more efficiently into mature, immunostimulatory DCs than SLE-derived moDCs (117–119).

In control tolDCs, modest downregulation of *BATF2*, an IFN-associated transcription factor, may help maintain a restrained activation state conducive to tolerance (120). Reduced BATF2 levels may limit pro-inflammatory BATF2–IRF4 complexes and instead favor IRF4-driven tolerogenic or homeostatic pathways (120). Consistent with this regulatory landscape, we observed mild upregulation of *STAT1* in control tolDCs. Given that *STAT1* is essential for DCs differentiation, maturation, and IFN-responsive regulatory circuits, its higher expression in controls suggests preserved IFN-driven homeostatic signaling, supporting balanced antigen presentation and immune regulation (121, 122). Interestingly, this regulatory balance appears altered in SLE, where both *in vitro* moDCs and tolDCs showed downregulation of *LAG3*, which could impair their capacity to deliver inhibitory signals to T cells and contribute to defective peripheral tolerance (72, 123). Together, these pathways indicate that SLE monocytes carry a priming imprint that biases DCs differentiation toward a partially activated and metabolically dysregulated phenotype.

To situate our findings within an established framework, we adopted the consensus “tolDC signature” defined by Robertson et al. derived from a comprehensive transcriptomic meta-analysis of diverse tolerogenic differentiation protocols. In the context of IL-10 induced tolDCs, 12 of the top 20 genes reported by Robertson et al. were concordantly differentially expressed in our tolDCs, supporting that our *in vitro* protocol captures many core features of IL-10 driven tolerogenic programming. Beyond these shared signature genes, our tolDCs also expressed markers associated with the human IL-10 producing DC-10 subset (e.g., *IL-10, CD163*) and exhibited a pattern of *CD80* upregulation with *CD83* downregulation consistent with semi-mature phenotypes under IL-10/IL-4 conditions. Together, these findings support that our differentiation protocol successfully recapitulates core aspects of IL-10–mediated tolerogenic DCs programming. The subset of oppositely regulated genes relative to the Robertson et al., analysis may reflect differences in differentiation timing or culture conditions. Notably, we observed selective upregulation of IL-7 in SLE-derived tolDCs, which may reflect the chronic inflammatory milieu of SLE and could contribute to enhanced DCs maturation and pro-inflammatory signaling, consistent with the established role of IL-7 in sustaining autoreactive immune responses in autoimmune disease (124).

Our findings reveal that the transcriptional programs governing DCs differentiation are profoundly reshaped in the context of SLE (125). By performing an interaction analysis, we identified disease-specific gene expression changes that distinguish *in vitro* moDCs and tolDCs from their healthy counterparts. The predominance of downregulated genes, particularly those involved in interferon signaling, antiviral responses, and immune regulation, underscores the extent to which SLE disrupts canonical pathways essential for DCs function (70, 126–130). The downregulation of negative regulators of IFN signaling in *in vitro* moDCs and tolDCs suggests a loss of feedback control mechanisms (131), which may perpetuate chronic interferon activity, thereby sustaining inflammatory programs and compromising tolerogenic differentiation in SLE DCs. Importantly, these SLE-specific alterations may be overlooked when tolDCs signatures are derived exclusively from healthy donors without considering disease context, highlighting the need to deepen the characterization of SLE-specific transcriptional programs to more accurately define tolerogenic DCs states in autoimmune settings.

Gene regulatory networks built from the transcriptome analysis of the differentiation of monocytes to DCs in SLE and controls revealed a shared transcriptional architecture shaping DCs differentiation with a regulatory signature in SLE. SLE DCs exhibited a higher number of unique regulons (n = 6 vs. 2 in controls), suggesting that SLE monocytes undergo DCs differentiation with a pre-existing regulatory bias, likely shaped by chronic inflammation and IFN signaling. One of the most prominent signals was the *BCL3* regulon, upregulated during *in vitro* moDCs differentiation (LFC = -0.05, p adj = 1.57x10^-3^). Our regulatory networks analysis highlighted the involvement of less-characterized zinc-finger/BTB transcription factors, particularly BCL11A and ZBTB7B, which have been recently implicated in regulating myeloid commitment, differentiation, and maturation in murine models (132). Although classically associated with T cell development, its significant activity in our human *in vitro* system suggests a potential role in shaping DCs development and homeostasis (132). The identification of BCL11A as the most strongly downregulated regulon in tolDCs, together with the enrichment of interferon-related target genes, supports a close association between BCL11A-linked regulatory activity and interferon-responsive transcriptional programs in the SLE context. Notably, the presence of USP18, ISG15, and IFIT family members among the differentially expressed targets, as well as the persistence of USP18 and ISG15 dysregulation from monocytes to tolDCs, points to a sustained interferon-associated regulatory state across differentiation stages (76, 133–135).

In contrast, although ZBTB7B exhibited the largest regulon-level activity difference between SLE and control tolDCs, its transcriptional footprint at the level of differentially expressed targets was limited, with only *KDM1A* (LSD1) identified in moDCs. While network-based analyses place ZBTB7B in proximity to genes involved in cholesterol transport, fatty-acid metabolism, and MHC-I antigen presentation, processes previously reported to be altered in SLE DCs (79), these associations are derived from co-expression patterns rather than direct regulatory evidence. As such, the functional involvement of ZBTB7B in these pathways should be regarded as hypothesis-generating and requires further experimental validation.

During tolDCs differentiation, additional regulators such as ZKSCAN1 and KLF16 were induced, whereas inflammatory drivers including RELA, BCL3, and IRF3 were downregulated (**Figure 3**), consistent with a coordinated program favoring a tolerogenic, non-inflammatory phenotype (136–142). Although these findings point to promising regulatory candidates involved in monocyte DCs differentiation, potentially altered in SLE, their roles remain correlative and require functional validation to confirm mechanistic relevance.

Although the transcriptional profile of our tolDCs resembles the well-described DC-10 phenotype, our study is limited by the absence of functional validation. Determining whether these *in vitro* generated cells recapitulate the immunosuppressive properties of bona fide tolDCs will require additional work. Future studies should include flow cytometric assessment of canonical DC-10 markers (CD141, CD163, HLA-G, ILT4/ILT receptors), quantification of IL-10 production and suppression of pro-inflammatory cytokines, and co-culture assays with naïve CD4_+_ T cells to evaluate their ability to induce regulatory T cells. Incorporating these functional assays will be essential to establish whether the observed transcriptomic programs translate into the tolerogenic activity expected of tolDCs and to refine our understanding of how these pathways are altered in SLE.

A limitation of this study is the lack of direct functional evaluation of tolDCs function by assessing cytokine production, T effector suppression, or Treg induction. While previous studies have shown that DCs generated under similar conditions exert tolerogenic functions, our conclusions are restricted to transcriptional and regulatory features associated with tolerance. Future work integrating functional assays will help to understand the contribution of these transcriptional profiles to immune tolerance dysregulation in SLE. Moreover, subtle differences between patients and controls along the differentiation trajectories may be attenuated by the highly controlled conditions of *in vitro* culture, which can partially normalize transcriptional states over time. For this reason, complementary single-cell and *ex vivo* approaches, applied closer to the time of sampling, may be better suited to capture disease-associated heterogeneity and transient regulatory states that are not fully preserved during prolonged *in vitro* differentiation.

## Conclusion

Our findings demonstrate an increased expression of interferon-stimulated genes in SLE patient monocytes relative to controls, thereby confirming a robust ISGs signature that persists as these cells differentiate into DCs subsets. Both *in vitro* moDCs and tolDCs exhibited disease specific transcriptional remodeling involving immune signaling pathways, lipid metabolism, and epigenetic regulators. The analysis of the tolerogenic signature revealed both concordance and divergence from canonical *in vitro* models, suggesting that tolerogenic programming is strongly shaped by the cellular and inflammatory context of SLE.

Although transcriptional differences in tolDCs between SLE and controls were generally subtle, the trajectory analyses indicate that key mechanisms required for establishing tolerogenic capacity may be disrupted in SLE. The persistence of interferon-driven programs across differentiation trajectories highlights the IFN-JAK/STAT axis as a central pathway influencing DCs biology in SLE, supporting the rationale for therapeutic strategies targeting interferon signaling such as anti-type I interferon biologics (e.g., anifrolumab) and JAK inhibitors (e.g., upadacitinib), In parallel alterations in metabolic and epigenetic regulatory pathways suggest additional mechanisms that may modulate tolerogenic function. Together, these findings highlight the importance of characterizing tolDCs heterogeneity and their regulatory networks to identify molecular pathways that could be therapeutically targeted to reinforce tolerogenic DCs function and ultimately help to restore immune tolerance in SLE.

## Data Availability

The datasets presented in this study can be found in online repositories. The names of the repository/repositories and accession number(s) can be found in the article/**Supplementary Material**.
