# Supplementary material for: Gene expression profiling of dendritic cell tolerance dysfunction in women with systemic lupus erythematosus
Source: Front Immunol. 2026 Jun 2;17:1771959. doi: 10.3389/fimmu.2026.1771959 (PMC13270180; doi:10.3389/fimmu.2026.1771959)
Supplement: Supplementary file 1 [file DataSheet1.docx]

**Supplementary information**

**Table S1**. Clinical and sociodemographic information per volunteer used for normality assessment and descriptive analyses. GCC: glucocorticoids, AM: antimalarials, MMF: mycophenolate mofetil, AZT: azathioprine, MTT: methotrexate, PDN: prednisone, PDS: prednisolone, Ctrl: non-SLE affected controls, SLE: Systemic lupus erythematosus.

**Table S2.** Sequencing and collection metadata of transcriptomic samples generated in the present study.

**Table S3.** Distribution statistics and normality testing results of continuous clinical variables using the Shapiro–Wilk test. SD: standard deviation, IQR: interquartile range.

**Table S4.** Clinical information per transcriptomic sample generated in the present study.

**Table S5.** Differential expression results from all comparisons between SLE and control samples. Comparisons were performed within each cell type. The cell type and expression direction per gene is indicated in the "Comparison_direction" column.

**Table S6.** Established SLE transcriptional signatures compiled from the literature.

**Table S7.** Differential expression results and enriched biological and REAC processes of Interferon Stimulated Genes (ISG) from the comparison between SLE and control samples. The column "Shared_expression_pattern" refers to the shared expression patterns for the corresponding gene across cell types: “Tri-lineage” denotes genes upregulated in monocytes, in vitro moDCs, and tolDCs. “Monocyte (UP)” refers to genes consistently upregulated in monocytes, while “moDCs (DOWN)” indicates genes consistently downregulated in in vitro moDCs. Data from this table was used for Figure 4.

**Table S8.** Correlation analyses between the normalized expression (z‑scores) of the 32 interferon‑stimulated genes (ISGs) identified as differentially expressed in our study and disease activity (SLEDAI), per cell type (monocytes, moDCs, and tolDCs), using Spearman correlation. Correlation was computed in each cell type separately, denoted in the column "cell_type"

**Table S9.** Pathway enrichment analysis results of all differentially expressed genes (DEGs) from comparisons between SLE and control samples within cell type. Data from this table was used for Supplementary Figure S4.

**Table S10.** Differential expression results from comparisons between moDC or tolDC against monocytes. Comparisons were performed within each sample group (SLE or Ctrl). The corresponding comparison and direction of expression per gene is shown in the "Comparison_direction" column.

**Table S11.** Previously reported differentiation-associated genes. The reported expression on the literature is shown in the "Expression" column. The differentiation process is denoted in the column "contrast".

**Table S12.** Biological processes linked to previously reported differentially expressed genes: concordant and divergent expression patterns. Whether our differential expression direction results are consistent or divergent from the expected according to literature is annotated in the "Category" column.

**Table S13.** Activity scores of regulons whose predicted gene targets overlapped with DEGs shared or unique to in vitro moDCs and tolDCs. Activity scores are measured by Scenic's normalized AUC values expressed as z-scores.

**Table S14.** Persistence of interferon-related gene expression programs from monocytes to dendritic cell differentiation. Overlap between differentially expressed genes in the monocyte SLE vs. control comparison and genes showing a significant interaction effect during differentiation.

# **Supplementary Figures**
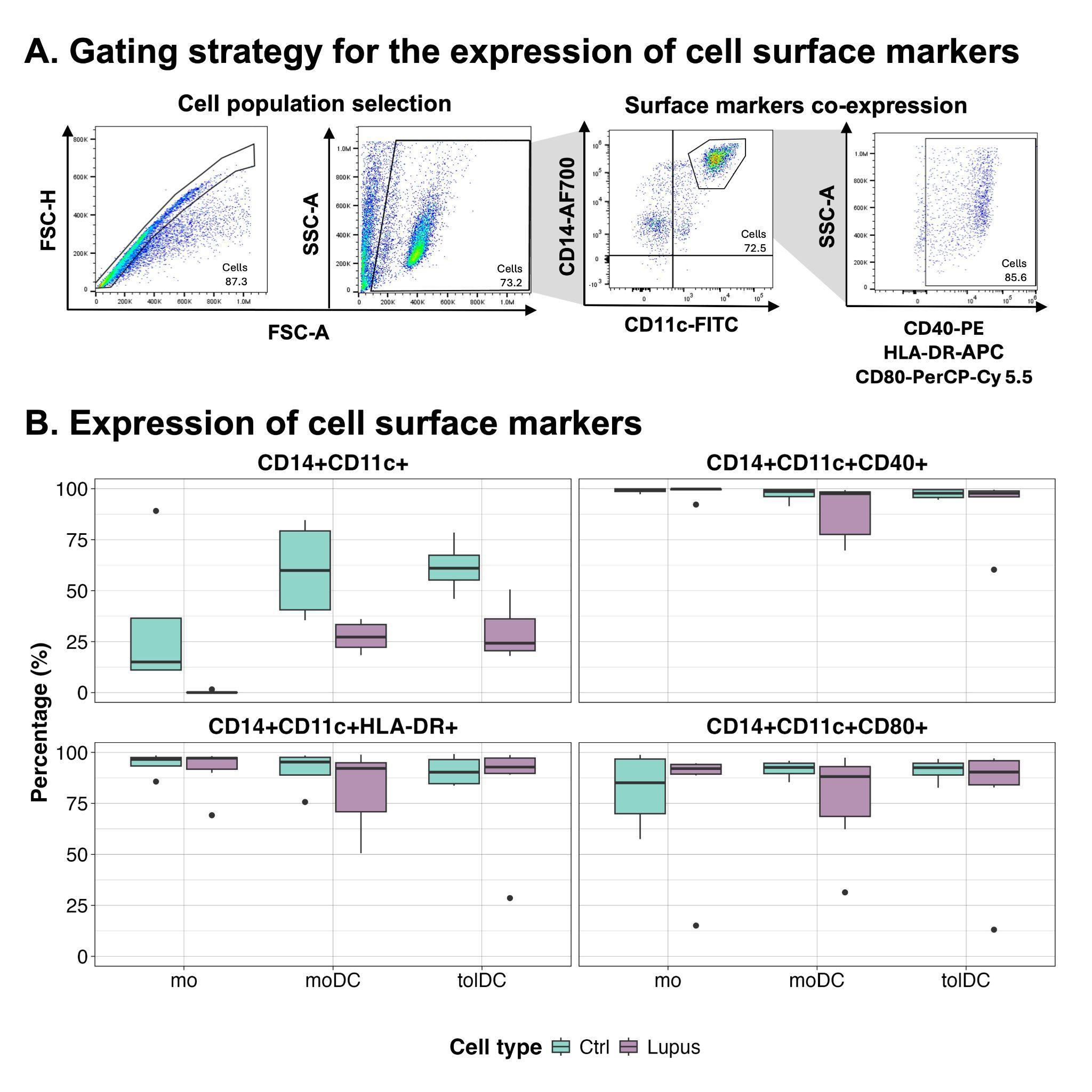


|  |
| --- |
| **Supplementary Figure S1. Flow cytometric analysis and quantitative bar chart analyses of surface marker expression. (A)** Gating strategy for the analysis of monocyte and dendritic cells surface marker expression. **(B)** Expression of CD14, CD11c, HLA-DR, CD80 and CD40 are shown among monocytes, monocyte-derived dendritic cells (moDCs), and tolerogenic dendritic cells (tolDCs) of people with SLE (purple bars) and controls (aqua bars). |

| **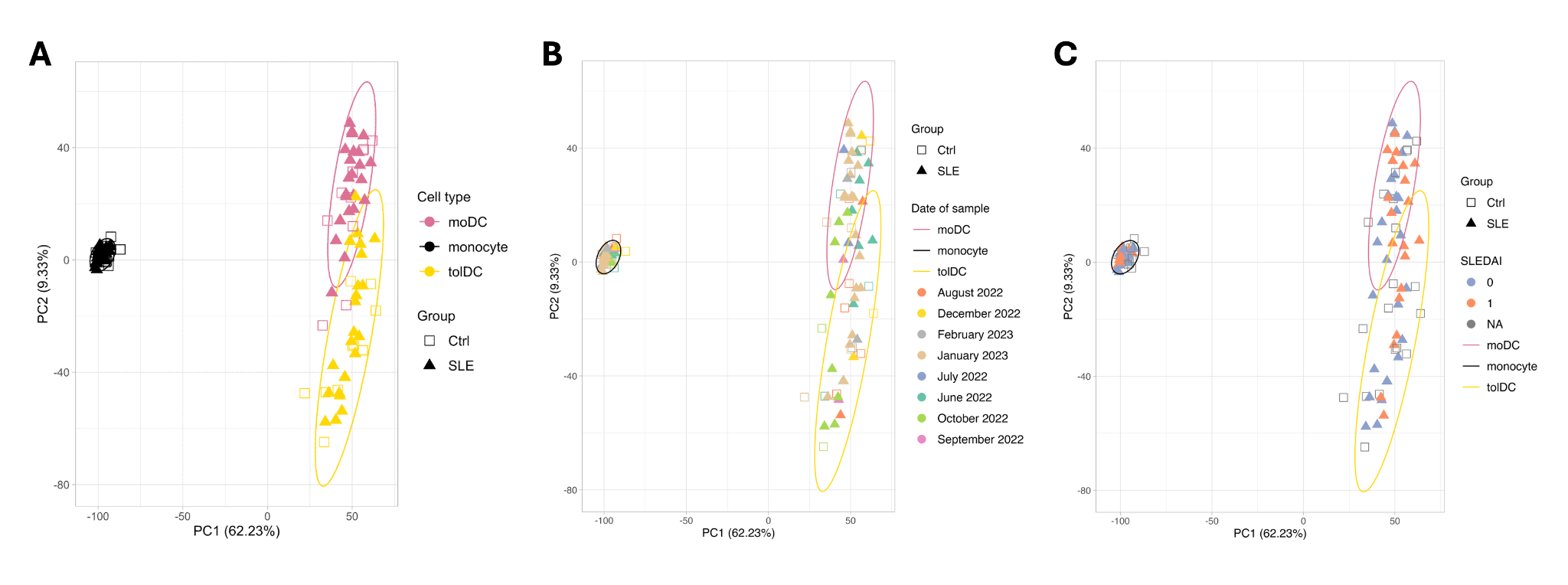** |
| --- |
| **Supplementary Figure S2. Principal Component Analysis (PCA) of normalized RNA-seq counts. (A)** PCA colored by Cell type (monocytes, moDC, and tolDC) and shaped by Group (SLE and Ctrl), showing clear segregation primarily driven by cell identity. **(B)** PCA plot including sampling dates (collection date) to evaluate potential batch effects across collections, revealing no major clustering by date. **(C)** PCA colored by SLEDAI disease activity, where 1 denotes a SLEDAI score > 0, 0 is no activity, controls are given NA. The first two principal components (PC1 and PC2) explain 62.23% and 9.33% of the total variance, respectively. Each point represents one sample. Abbreviations: SLE, systemic lupus erythematosus; Ctrl, healthy control; mo, monocytes; moDC, monocyte-derived dendritic cells; tolDC, tolerogenic dendritic cells. The data underlying this figure can be found in Supplementary Table S1 and Dataset S2. |

| 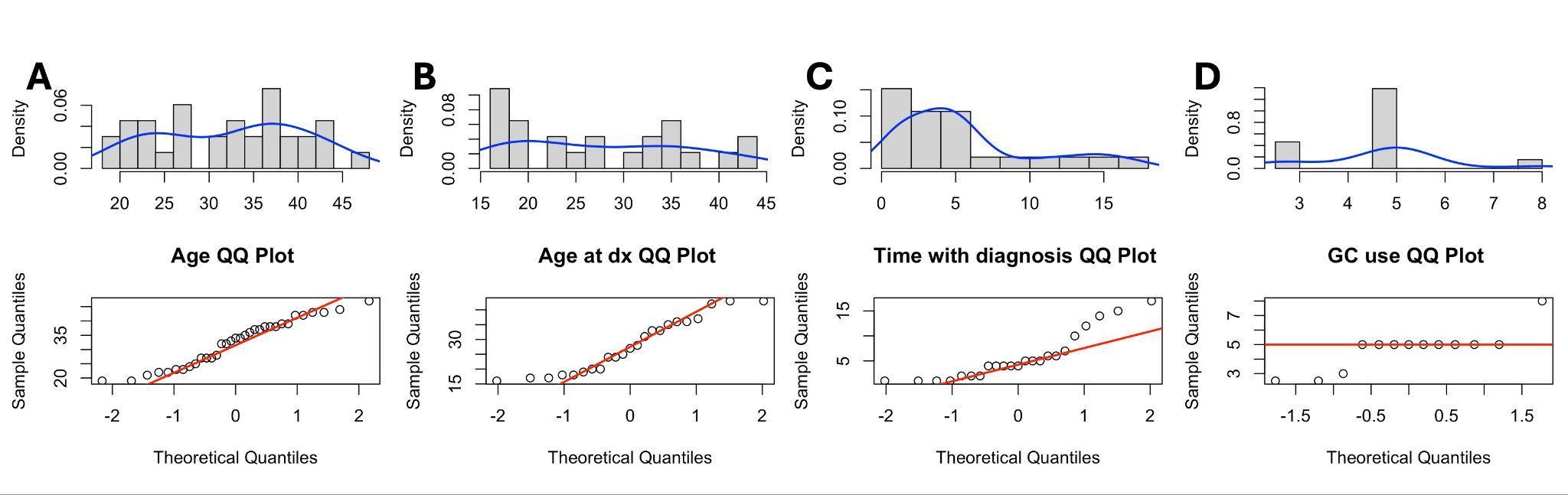 |
| --- |
| **Supplementary Figure S3. Distribution and normality assessment of continuous variables.** Barplots are divided into panels showing density distributions and Q–Q plots for **(A)** Age, **(B)** Age at treatment initiation, **(C)** Time since diagnosis, and **(D)** corticosteroid use **(QC).** Panel **(E)** displays the scatterplot of sample quantiles versus theoretical quantiles. These plots illustrate the distributional properties of the variables and their deviation from normality, as assessed by Shapiro–Wilk tests.  **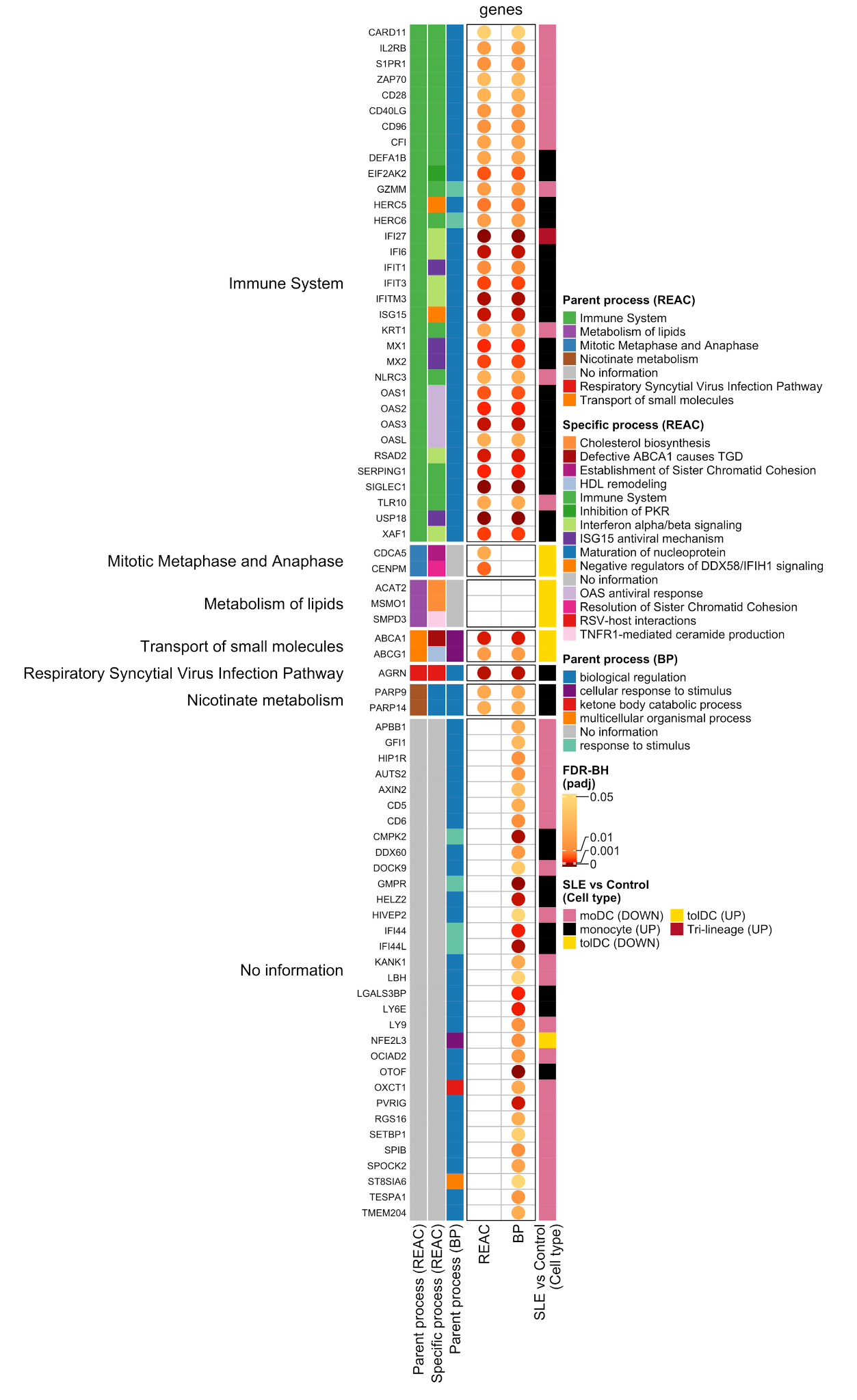** |

**Supplementary Figure S4. Biological processes involved and associated with the DEGs obtained by comparing SLE vs. Ctrl in each cell type.** Processes were classified according to Reactome (REAC) parent and specific categories, and Gene Ontology Biological Process (GO:BP) parent terms. REAC parent processes include Immune system, Synthesis of phosphatidylinositol (PI), Cell division, Lipid metabolism and Transport of small molecules.

Color scale represents padj (FDR–BH correction), with a maximum threshold of padj < 0.05. DEGs: Differential expressed genes. The data underlying this figure can be found in Supplementary Table S6.


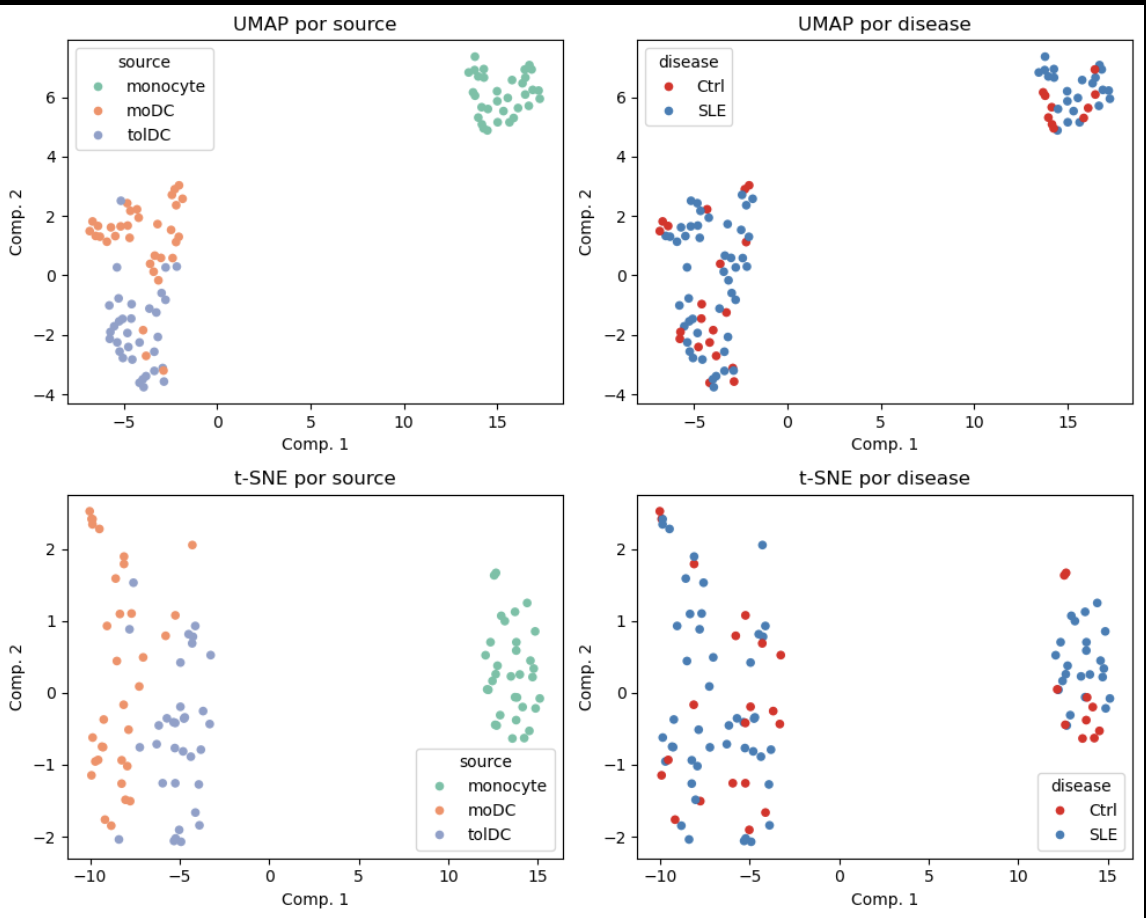


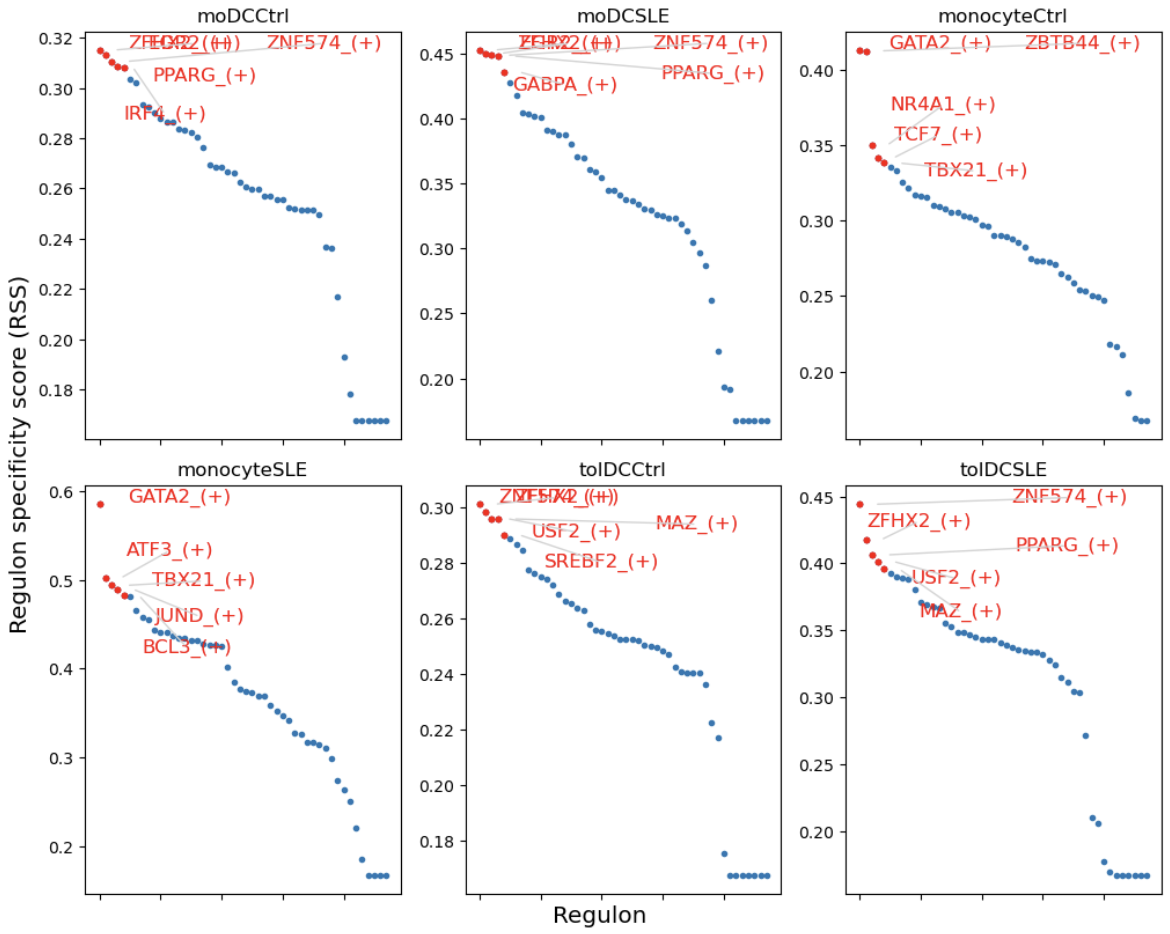


**Supplementary Figure S5. UMAP visualization and regulon specificity analysis of monocyte-derived populations. (A)** UMAP projection based on components 1 and 2, colored by cell type (monocytes in green, moDC in orange, and tolDC in blue), showing clear segregation of samples according to cell identity. **(B)** UMAP projection of the same dataset, colored by group (SLE in blue and Ctrl in red), illustrates the distribution of samples across disease conditions. **(C)** Regulon Specificity Score (RSS) analysis highlighting the top five transcription factors (TFs) identified with pySCENIC. The knee plot depicts the ranking of regulons by specificity score. The data underlying this figure were derived from the file *multi_runs_regulons_auc_trk.loom* and are available at Zenodo, DOI: 10.5281/zenodo.17419882.


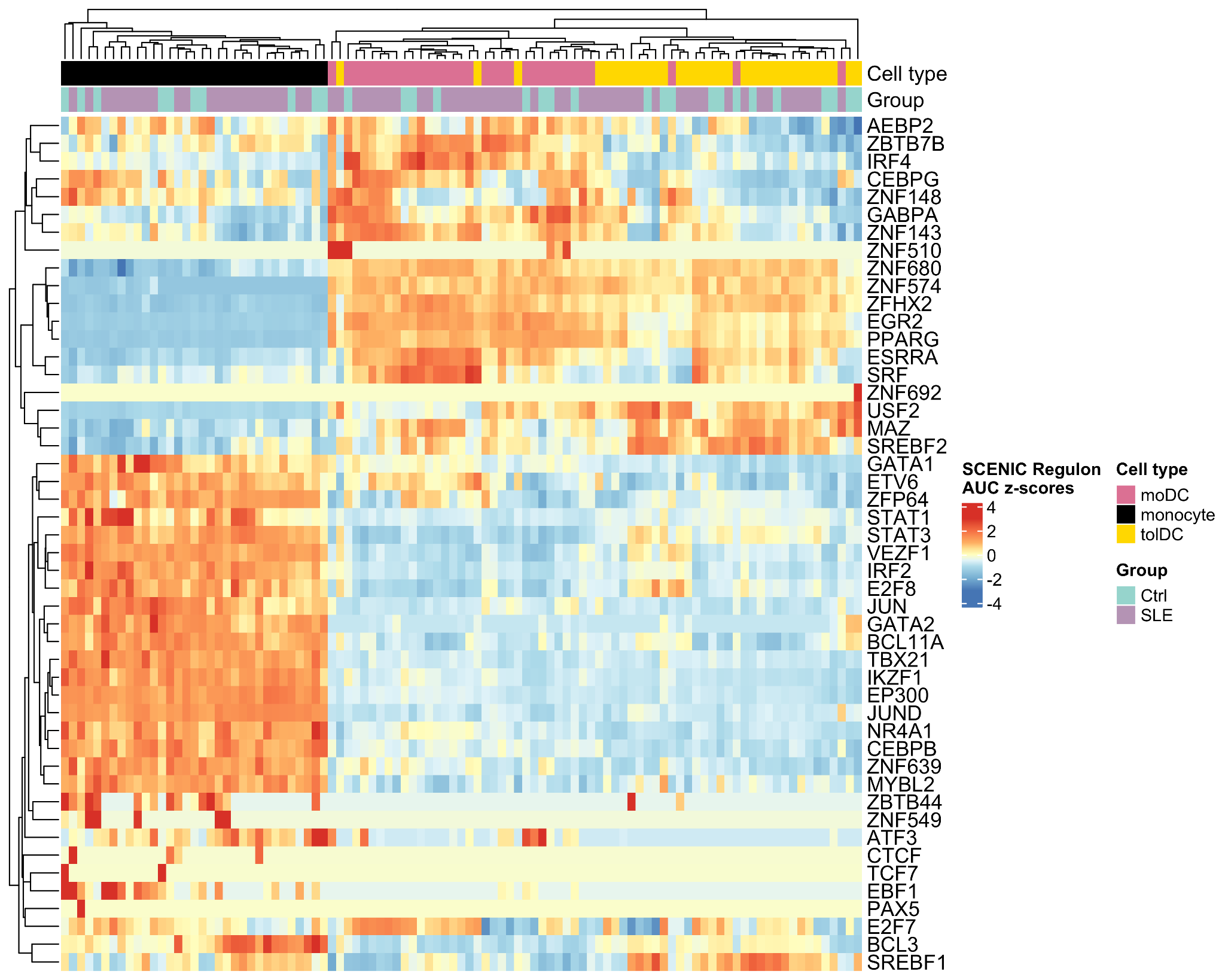


**Supplementary Figure S6. Regulon activity scores (AUC, z‑score normalized).** Heatmap representation of regulon activity across samples, showing normalized AUC values expressed as z‑scores. Rows correspond to individual regulons, while columns represent samples according to conditions (SLE or Ctrl). Color intensity reflects relative activity, highlighting the regulons with increased or decreased activity patterns. The data underlying this figure can be found in Supplementary Table S14.


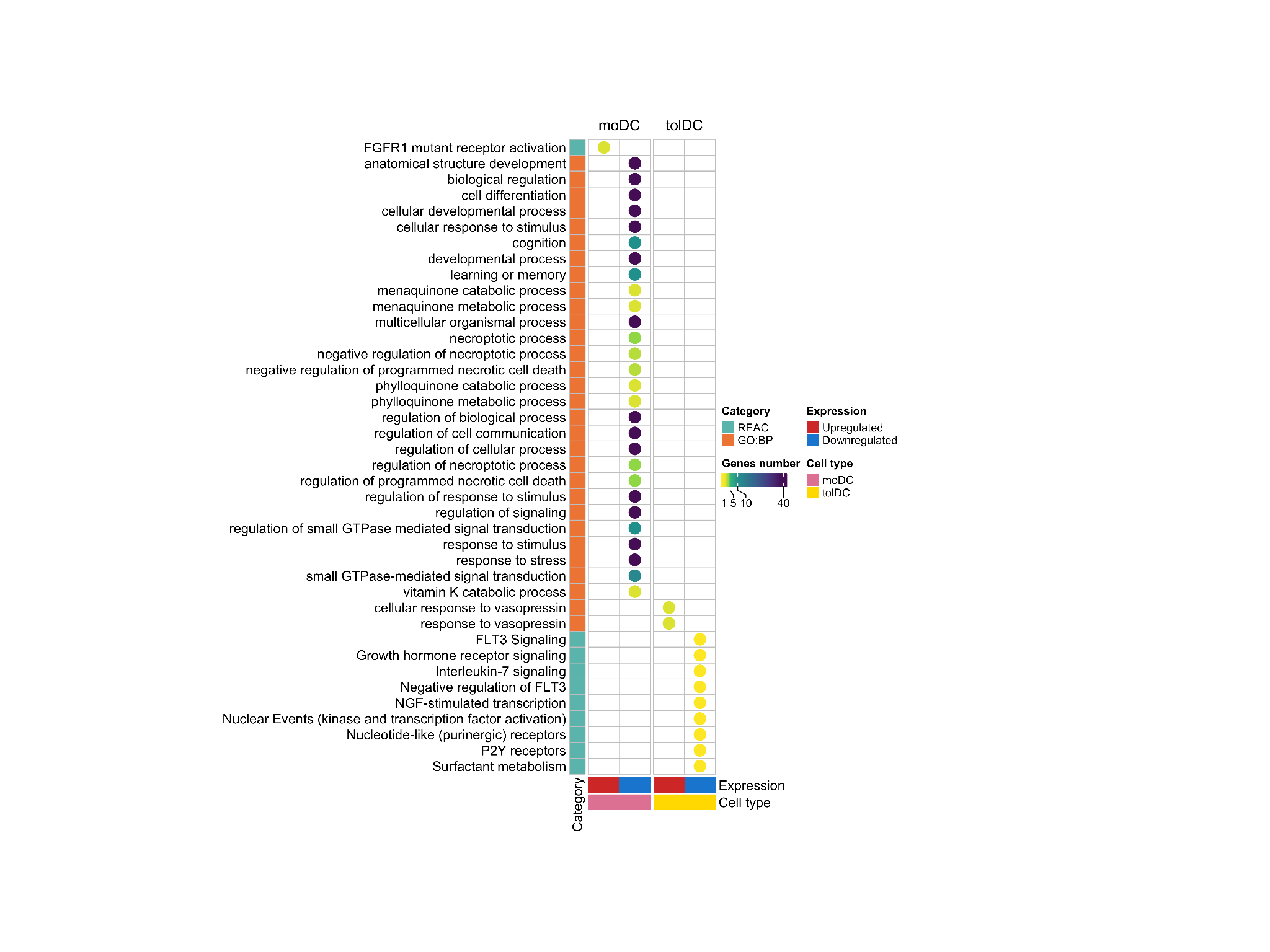


|  |
| --- |
|  |

**Supplementary Figure S7. Gene enrichment analysis of SLE-exclusive regulons involved in the differentiation to moDC and tolDC.** Terms from the Gene Ontology Biological Process (GO:BP) and Reactome (REAC) pathway categories enriched among the target genes of moDC and tolDC SLE-specific regulons. These target genes were also significantly differentially expressed during the differentiation process. Terms are listed in rows. Each term’s category (REAC or GO:BP) is annotated on the left. Columns indicate the cell type associated with each regulon (annotated as “Cell type”). The “Expression” annotation denotes whether each regulon set was up or downregulated during differentiation to the corresponding cell type. The color scale represents the number of differentially expressed target genes associated with each term.

|  |
| --- |
|  |

| 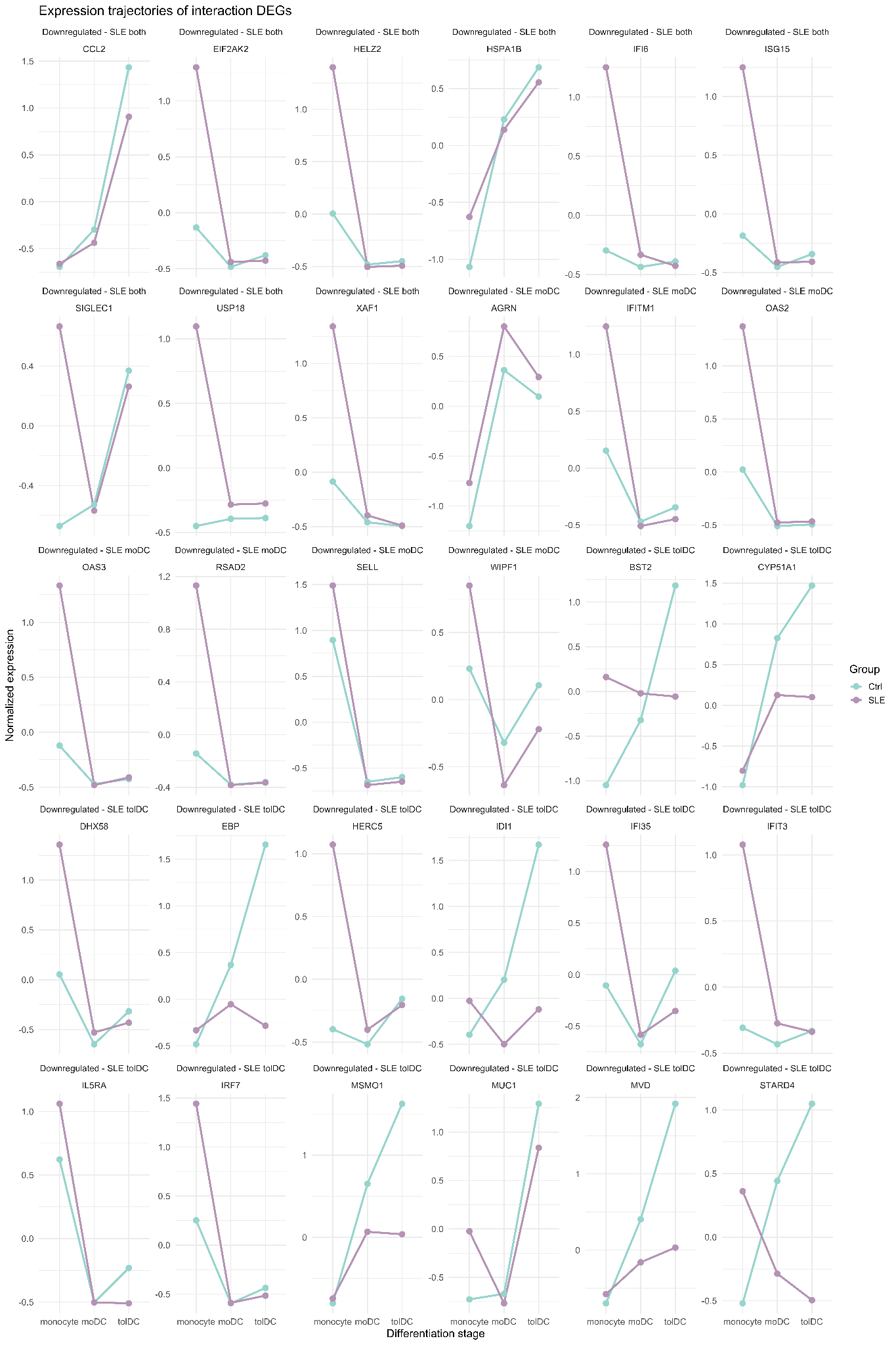 |
| --- |
|  |

| **Supplementary Figure S8. Expression trajectories of 30 downregulated interaction DEGs across differentiation stages (Monocyte to moDC, and to tolDC) in controls (Ctrl) and SLE.** Lines represent mean normalized expression (z‑score) per group, with points indicating each differentiation stage. Genes are grouped according to their classification (Downregulated – SLE both, Downregulated – SLE moDC, Downregulated – SLE tolDC). The trajectories highlight that most interaction DEGs show attenuated induction in SLE compared to controls, consistent with disease‑specific modulation of dendritic cell differentiation. |
| --- |
| 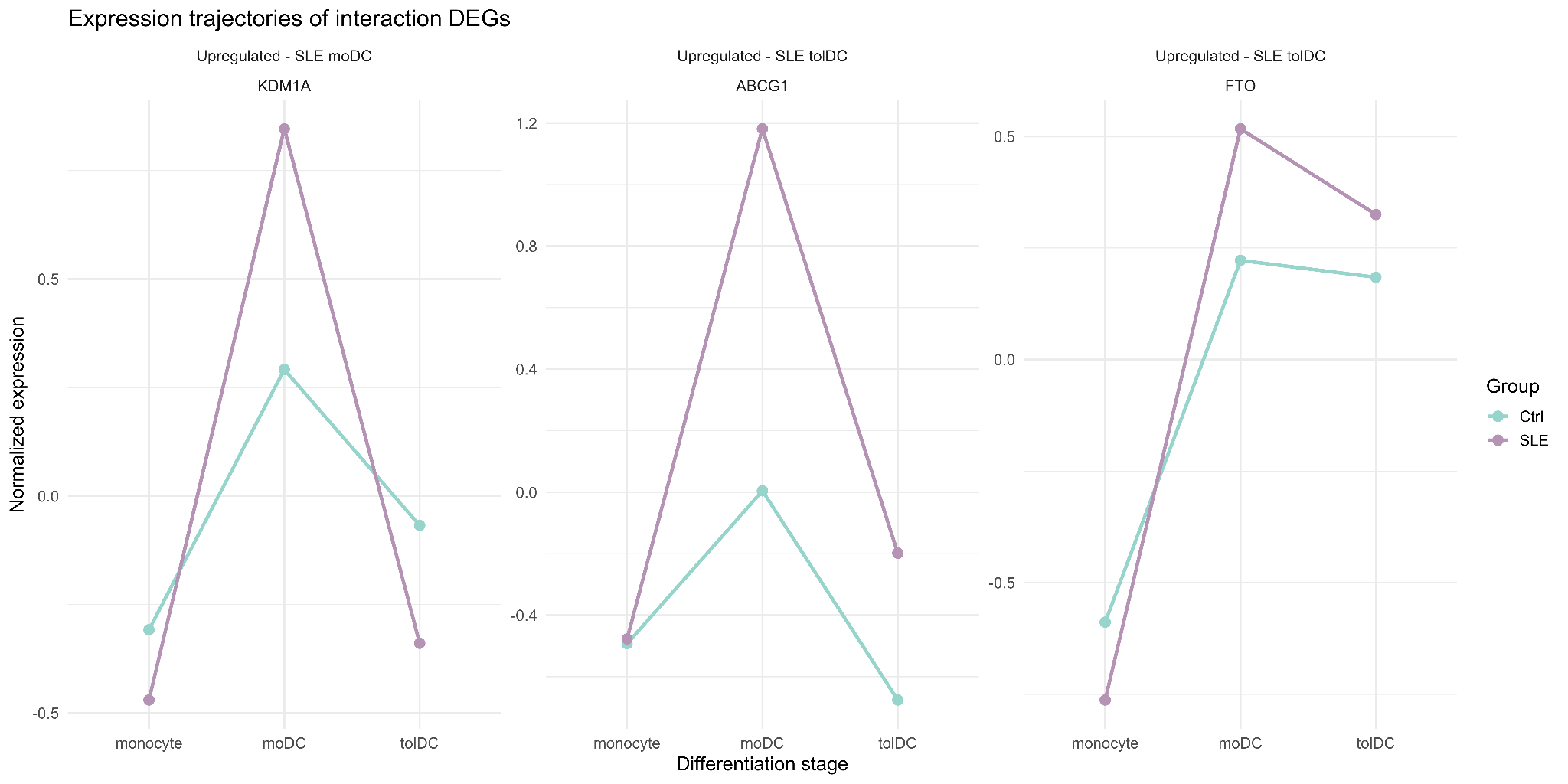  **Supplementary Figure S9. Expression trajectories of 3 upregulated interaction DEGs across differentiation stages (Monocyte to moDC, and to tolDC) in controls (Ctrl) and SLE.** Lines represent mean normalized expression (z‑score) per group, with points indicating each differentiation stage. Genes are grouped according to their classification (Upregulated – SLE both, Upregulated – SLE moDC, Upregulated – SLE tolDC). The trajectories highlight that these 3 interaction DEGs show enhanced induction in SLE compared to controls, consistent with disease‑specific modulation of dendritic cell differentiation. |

| 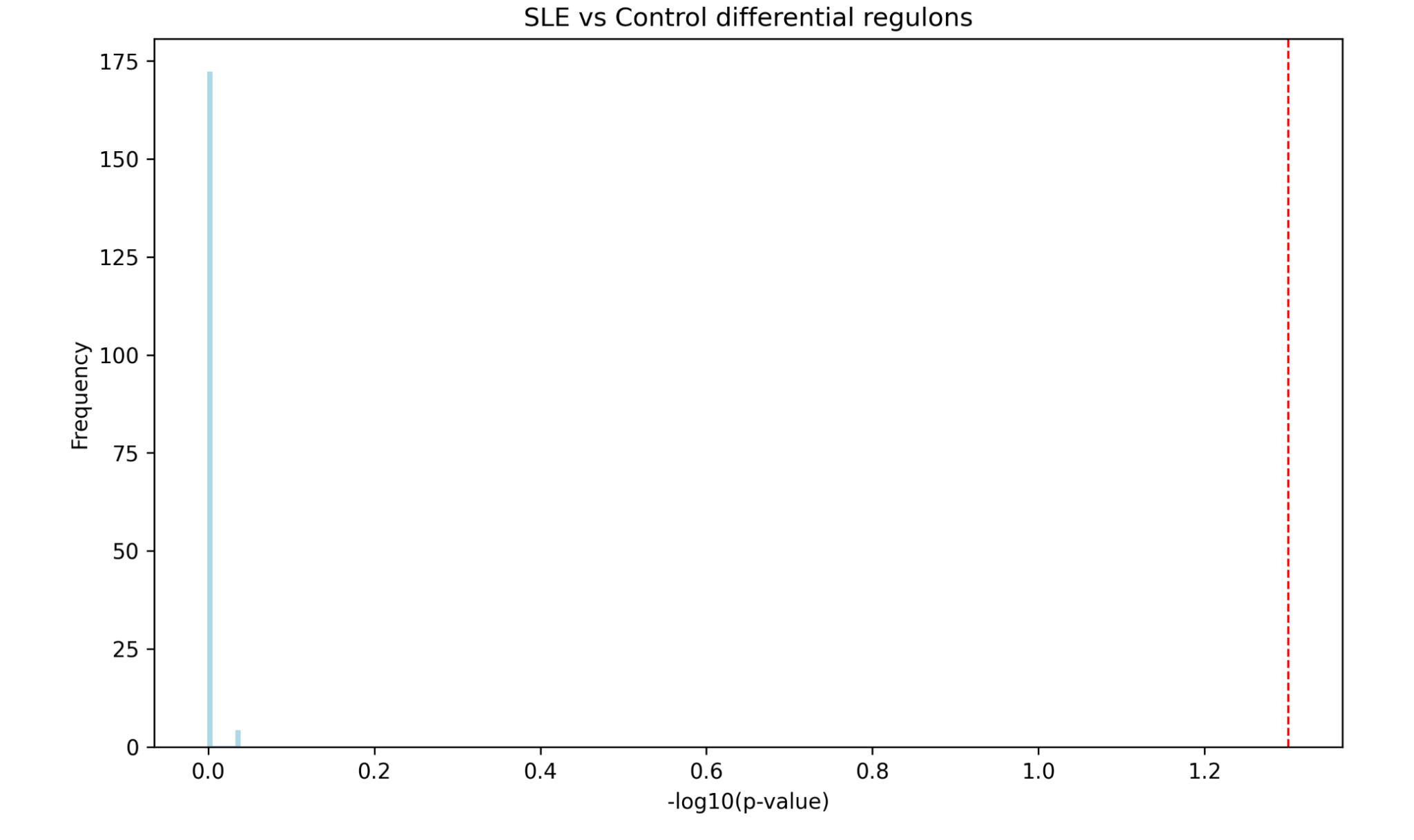 |
| --- |
|  |

**Supplementary Figure S10.** Differential regulons adjusted p-values from the SLE vs control comparison

Histogram of p-values from the differential analysis of regulons in SLE patients compared to controls. P-values were adjusted for multiple testing using the Benjamini-Hochberg procedure (FDR < 0.05) and -log10 transformed. The red line shows the significant cut-off threshold at 0.05.

| 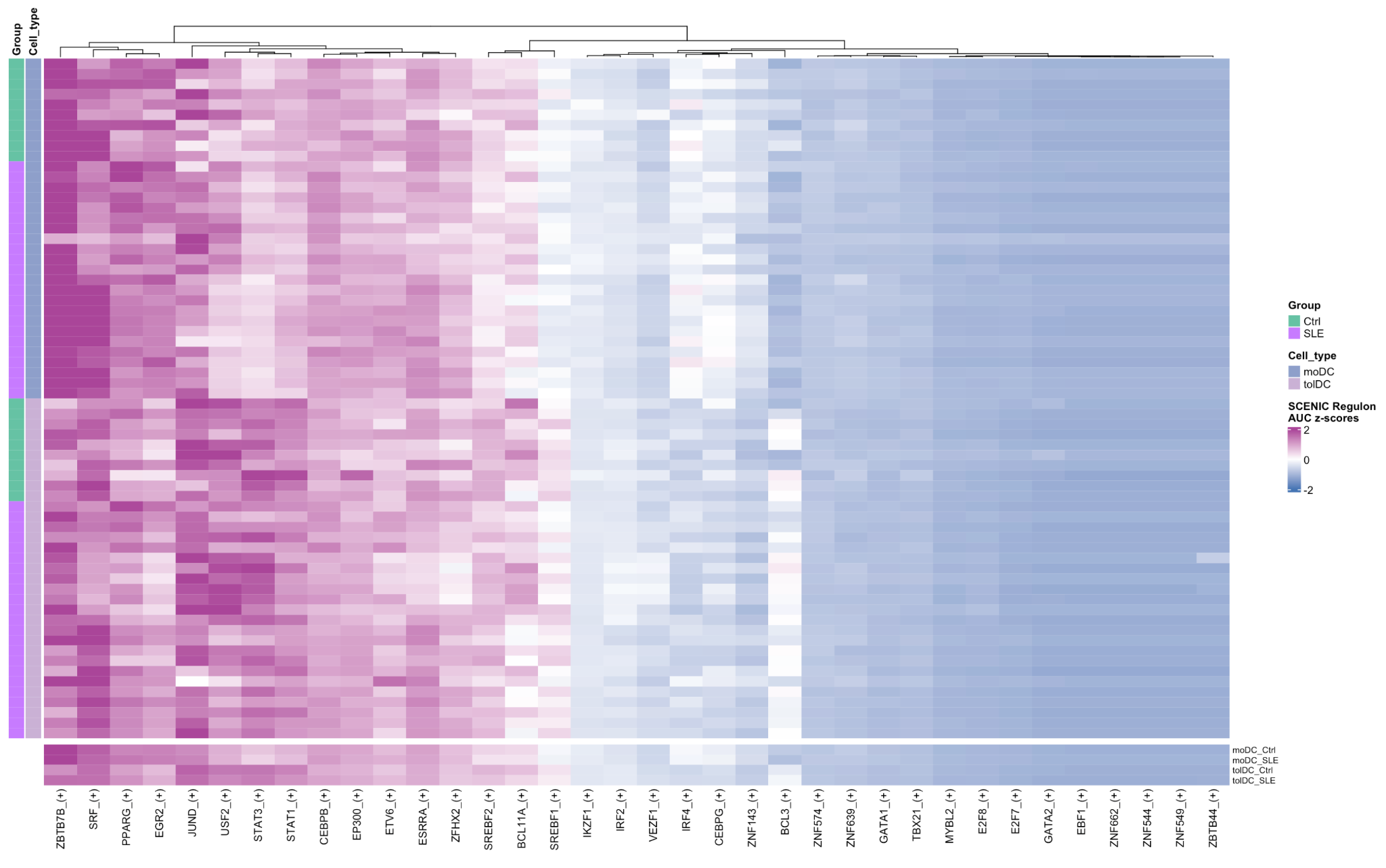 |
| --- |
|  |

**Supplementary Figure S11. Regulon activity restricted to DEGs of interest (shared DEG from Figure 6A).**

Activity of regulons that contain at least one differentially expressed gene (DEG) of interest among their targets in moDC and tolDC from SLE and control samples. For each regulon, we show the distribution of AUC z-scores across conditions.

| 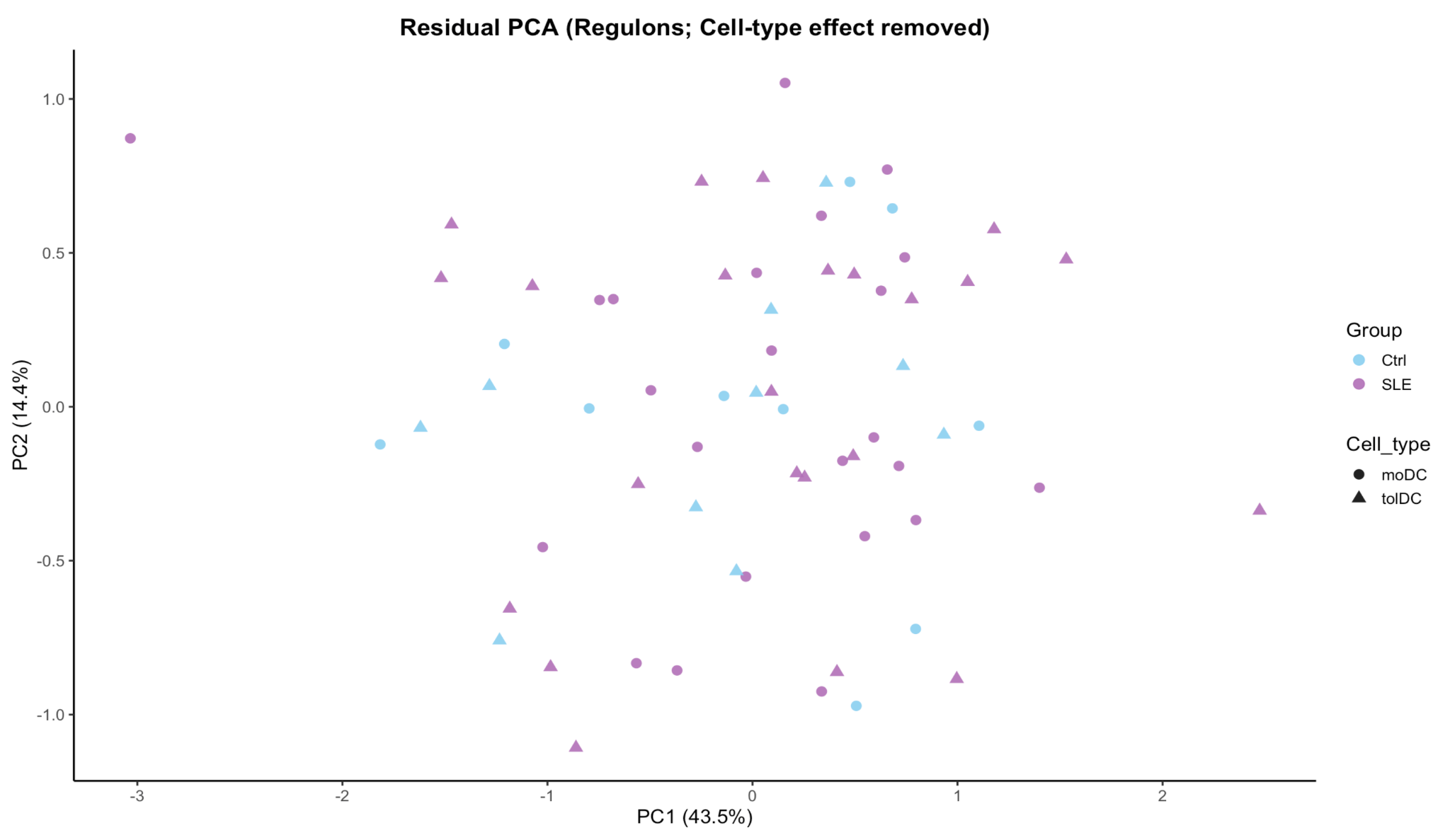 |
| --- |
|  |

**Supplementary Figure S12. Residual PCA of regulon activity after removing cell-type effects**

Residual principal component analysis (PCA) of regulon activity after regressing out the effect of cell type from the AUC matrix using a linear model. In the original PCA using the full set of DEGs, the main axis of variation was driven almost exclusively by cell identity, with no clear segregation between SLE and control samples. The residual PCA, computed on regulon AUC scores after removing the cell-type effect, similarly failed to reveal a distinct separation by disease status. These results indicate that the dominant structure of the regulon landscape is shaped by cell differentiation state (moDC vs tolDC), and that any disease-associated differences occur as more subtle shifts in specific regulons rather than as a global reorganization of regulatory programs.

| 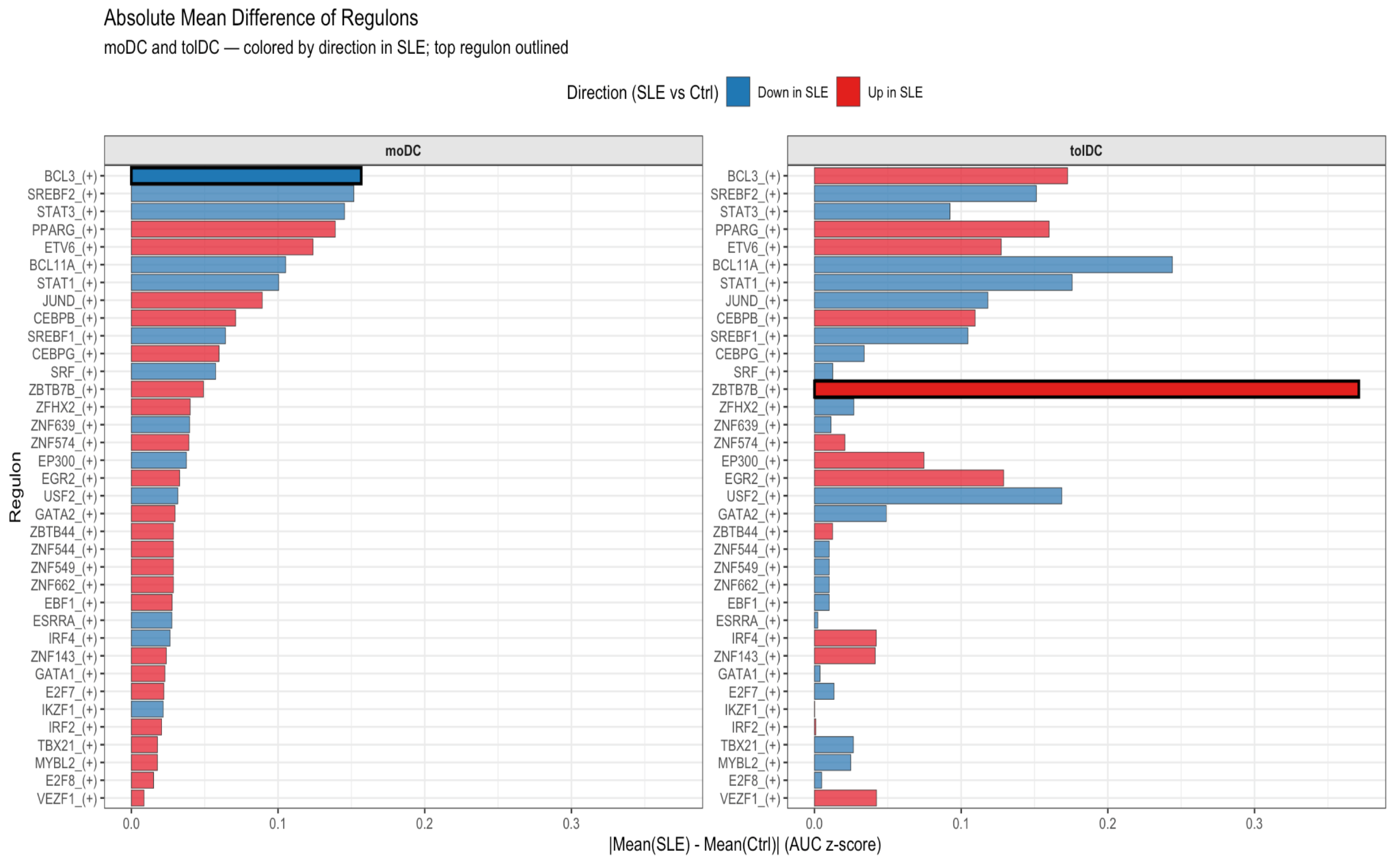 |
| --- |
|  |

**Supplementary Figure S13. Absolute difference in regulon activity between SLE and controls**For each regulon, the absolute difference in mean AUC z-score between SLE and control samples, computed separately for moDC and tolDC. This metric captures the magnitude of condition-specific changes in regulon activity while ignoring directionality, thereby allowing the identification of regulons that are most strongly perturbed in SLE, those being up-regulated are highlighted in red and those down-regulated are highlighted in blue . Within this framework, ZBTB7B emerges as the regulon with the largest difference in tolDCs and BCL2 within moDCs.
